# Study protocol for a controlled clinical trial on cardiac telerehabilitation and educational follow-up in patients with heart failure

**DOI:** 10.1371/journal.pone.0327366

**Published:** 2025-07-10

**Authors:** Jhonatan Betancourt-Peña, Iago Portela-Pino, María José Martínez-Patiño

**Affiliations:** 1 Faculty of Health Rehabilitation, Institución Universitaria Escuela Nacional del Deporte, Cali, Colombia; 2 Department of Health Sciences, Department of Didactics and Theory of General and Specific Education, University of León, León, Castilla y León, Spain; 3 Faculty of Education and Sports Sciences, Universidad de Vigo, Vigo, Spain; University of Chile: Universidad de Chile, CHILE

## Abstract

**Background:**

Heart failure is a primary non-communicable disease in Latin America and the Caribbean, which significantly reduces patients’ quality of life. After diagnosis, emphasis is placed on cardiac rehabilitation and patient education regarding healthy lifestyles and habits. Interdisciplinary management is crucial in this process. However, lack of treatment adherence is a recurring issue in this population. In this context, technology allows us to address non-adherence to treatment. Its implementation helps overcome barriers and achieve treatment goals.

**Objective:**

To determine the effects of a cardiac telerehabilitation-mediated exercise program and telephone educational follow-up on functional capacity in patients with heart failure, compared with cardiac telerehabilitation and conventional outpatient cardiac rehabilitation.

**Methods:**

This article presents a SPIRIT-compliant protocol study of a three-arm, controlled, crossover clinical trial using participant blinding and simple sealed envelope block randomization.

**Conclusion:**

According to the literature review, this can be considered the first three-arm clinical trial in the telerehabilitation of patients with heart failure. The measurement and intervention instruments are comparable and adequate compared to other studies.

**Trial registration:**

ClinicalTrials.gov NCT05761639

## Introduction

Cardiovascular diseases (CVDs) are a significant public health problem worldwide and are considered the leading cause of preventable illness, disability, and mortality [1]. In Latin America and the Caribbean, CVDs are the leading cause of death from chronic non-communicable diseases. There are about 726,000 deaths per year, and these figures are increasing, being one of the leading causes of years lost in people [1]. Heart failure (HF) is one of the CVDs, so the incidence, prevalence, decline in quality of life, frequent hospitalizations, morbidity, and mortality associated with this disease are high, and chronic patients often present with disease-related decompensation [2,3].

In this regard, one of the best recommendations or Class I recommendations from scientific associations is cardiac rehabilitation (CR), which significantly improves adherence to treatment goals and survival in patients with HF [4,5]. CR programs improve quality of life and functionality and reduce long-term mortality by 20% to 30%. However, only 7.5% to 29% of patients are referred to and enrolled in a CR program, and only 40% to 55% of these patients can adhere to and complete treatment [6,7].

Lack of treatment adherence related to lifestyle changes and self-care is a factor that contributes to the occurrence of episodes of decompensation and hospitalization. This is reflected in the biomarkers and aerobic capacity measured through the 6-minute walk test [8]. Additionally, Poor health education, depressive symptoms, cognitive deficits, and multiple comorbidities may limit this low self-care capacity [6,9]. As a result, the low health literacy of HF patients translates into less knowledge about the disease, less self-care behaviors, deterioration of quality of life, and poor adherence to pharmacological and therapeutic treatment [9].

## Objective

The primary objective of this clinical trial is to assess the effects of a cardiac telerehabilitation-mediated exercise program combined with telephone educational follow-up on functional capacity in patients with HF. This will be compared to traditional outpatient cardiac rehabilitation.

The secondary objectives include evaluating the impact of the same telerehabilitation program on clinical variables, anxiety and depression, and health-related quality of life in HF patients, again in comparison to conventional outpatient cardiac rehabilitation. This study is designed as a three-arm randomized controlled trial, with blinding for the assessors, investigators, and data analysts.

## Materials and methods

### Study environment

**Fig 1:**
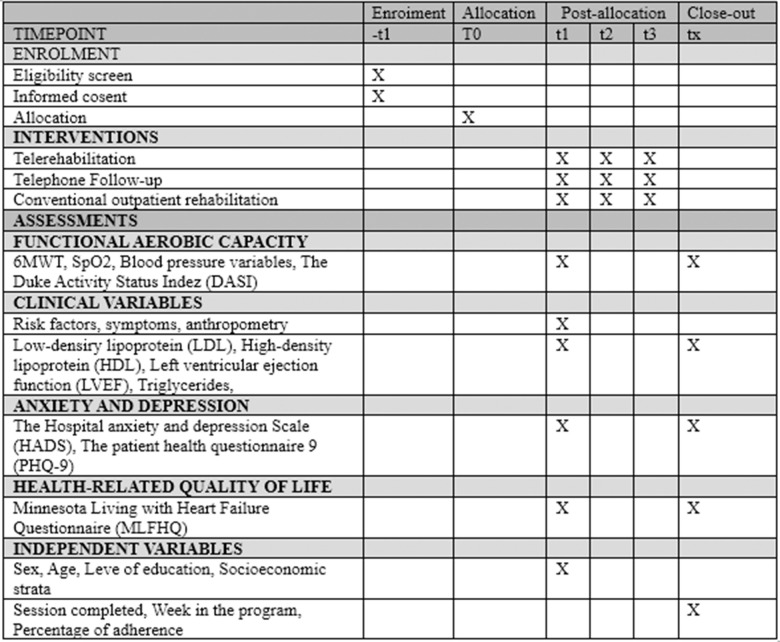
SPIRIT schedule of enrolment, interventions, and assessments. 6MWT: the 6-minute walk test.

This protocol study is a randomized controlled clinical trial of three-arm blinded to the assessor, the investigator and the information analyst. It has been registered on ClinicalTrials.gov (NCT05761639) and is currently collecting data, following the recommendations of the SPIRIT guide [9] (Fig 1). The study schedule is as follows: start was 15/10/2022, participant recruitment ended on 30/01/2025, and the estimated completion of the study is planned for 20/06/2025.

All patients will be informed about the study before recruitment. It will be conducted at the *Clínica de Occidente* SA in Cali, Colombia, where participants will be evaluated. Based on the evaluation, exercise prescriptions will be issued to guide patients during cardiac telerehabilitation and conventional outpatient cardiac rehabilitation.

### Ethics

This study adopted the ethical principles of the Declaration of Helsinki and Resolution 008430 of 1993 of the Ministry of Health and Social Security of Colombia. It was approved by the Ethics Committee of *the Clinica de Occidente S.A*. in June 2022 with code IYECDO-1598. All participants will be informed about the study, have their concerns addressed, and sign the informed consent form.

### Sample

The population will include patients with a diagnosis of HF who will be admitted to a cardiac rehabilitation program in the city of Santiago de Cali, Colombia. It should be noted that this study aims to compare quantitative variables. Studies that compare means use the standard deviation as a measure of variation, so the variable of interest will be the distance covered in meters in the 6-minute walk test (6MWT).

The hypothesis of this study proposes that the group receiving telerehabilitation, telephone follow-up, and education will achieve better results in patients’ aerobic capacity, compared to the other experimental groups.

There are no studies with similar characteristics with telephone follow-up to obtain a walking distance equal to that of the telerehabilitation group. This is because the groups are homogeneous at the beginning of the intervention but heterogeneous at the end of the intervention program; 378.5 ± 96.9 for the conventional outpatient cardiac rehabilitation group and 432 ± 83 for cardiac telerehabilitation).

In turn, 95% confidence is estimated, error α: 5% (0.05), error β: 20% (0.20), power: 1-β: 80%, giving n = 46 participants for each experimental group, which is increased by 12% for possible problems in the measurements or dropouts of the study participants, so that n = 52 will be required and a total of 156 patients with HF per experimental group at the end of the study.

### Criteria for eligibility

Participants aged 18–80 years of both sexes.

#### Inclusion.

- Patients previously diagnosed with HF by a cardiologist using the European Society of Cardiology (ESC) recommendations [4].
- Patients with an indication for exercise.
- Patients with electronic devices such as computers, tablets, or mobile phones.
- Participants who accept and sign the informed consent form.

#### Exclusion.

- Patients with respiratory comorbidity or any limitation to perform active and resistive movements (recent fractures, recent hemodynamic changes, coronary artery disease event after diagnosis of cardiovascular disease, infectious diseases, and neuromuscular limitations).

### Outcomes measures (dependent variables)

#### Functional aerobic capacity.

The distance covered in the 6MWT will be taken into account [10,11], with distances between <350m and <200m considered as risk factors [12]. It will be performed in a 30 m corridor with two cones at the ends to define the distance to be walked. Before the start, each patient will be stimulated to walk as fast as possible. The remaining time will be displayed for each minute elapsed, Therefore, two attempts of this test will be conducted, and the most significant distance, meaning the greatest distance, will be recorded. Participants will have a rest period between each attempt to return to their baseline state before starting the test again. Considering SpO2, HR, and blood pressure variables. The latter will be measured with a sphygmomanometer and aneroid sphygmomanometer (WelchAllyn® DS44−11CBT).

The 1-minute sit-to-stand repetitions will be performed with the patient seated. From an upright position in a chair against a wall at a standard height of 46 cm without armrests, the patient will have to stand and sit for 1 minute as quickly as possible [13]. The Duke Activity Status Index (DASI) functional capacity questionnaires [14] will be administered, with a score of <4 considered as a risk point [15].

#### Clinical variables.

These will be obtained from the initial measurements at enrollment in the CR program, a structured interview, and the patient’s medical history. Risk factors, symptoms, anthropometry (body mass index, abdominal circumference, fat percentage, water percentage, lean mass), left ventricular ejection fraction (LVEF), total cholesterol, high-density lipoprotein (HDL), low-density lipoprotein (LDL), triglycerides, and reason for leaving the program will be taken into account. The accepted values in cardiovascular patients of total cholesterol <200mg/dl, LDL < 100 mg/dl, HDL in men > 40 mg/dl, women >50 mg/dl, and triglycerides 150 mg/dl will also be considered [16].

#### Anxiety and depression.

The Hospital Anxiety and Depression Scale (HADS) is a self-administered instrument that measures symptoms of depression and anxiety in people without a psychiatric diagnosis. The scale score ranges from 0 to 21. It is scored on a Likert scale from 0 to 3: the higher the score, the greater the intensity or severity of symptoms [17]. The Patient Health Questionnaire 9 (PHQ-9) is a questionnaire that measures the presence and severity of depressive symptoms. It is widely used in primary care settings. It consists of the nine symptoms of depression according to the DSM-IV20 criteria [18].

#### Health-related quality of life.

HRQoL will be assessed using the Minnesota Living with Heart Failure Questionnaire (MLFHQ). It is an instrument used to know the quality of life of patients with HF and provides information for two dimensions, physical and emotional, in addition to the total score [14].

#### Independent variables.

The variables considered will be age, sex, marital status, health system, occupation, level of education, and socioeconomic strata (in Colombia, strata 1, 2, and 3 correspond to low strata, which include users with fewer resources who are beneficiaries of public utility subsidies. Strata 4, 5, and 6 correspond to high strata, which include users with more significant economic resources), sessions completed, weeks in the program, and percentage of adherence.

### Assignment of intervention

Intervention allocation will be randomized using the block and sealed envelope method. The investigators will prepare the envelopes. After patients have consulted with the specialist, a person unaffiliated with the research will ask them to select one of the envelopes for subsequent planning of the intervention process. This will maintain blinding of the assessors and investigators.

If a patient assigned to telerehabilitation drops out, unmasking will occur. As a result, a place in the experimental group to which the patient belonged will become available, and the patient will continue the rehabilitation sessions as usual. This will be the case for those assigned to telerehabilitation.

### Intervention

Prior to enrollment, patients will be seen in person for assessment of anthropometric data (height, weight, percent fat, water, muscle mass, and abdominal circumference), laboratory tests (total cholesterol, HDL, LDL, triglycerides, blood glucose, BUN), functional aerobic capacity (6MWT and sit-to-stand test), depression and anxiety questionnaires (HADS, Patient Health Questionnaire 9, PHQ-9), functional capacity (Duke Activity Status Index (DASI)), and health-related quality of life (HRQoL) Minnesota Living with Questionnaire MLFHQ [6]. These data will be collected again at the end of the 12-week intervention program.

The following is a detailed description of each intervention to be performed in this study, the assignment of the intervention according to the experimental group (Table 1), and the exercise prescription for each group (Table 2).

**Table 1:** Interventions assigned to each experimental group.

Arms	Intervention			
	Conventional rehabilitation	Education	Telerehabilitation	Telephone follow-up
Experimental Group 1		**x**	**x**	**x**
Experimental Group 2	**x**	**x**		
Experimental Group 3		**x**	**x**	

**Table 2:** Exercise prescription.

Arms	Exercise prescription
Experimental Group 1: Telerehabilitation, Education, and Telephone follow-up Experimental Group 3: Telerehabilitation and Education	**Devices:** Polar FT4 heart rate monitor to record heart rate and OMRON digital sphygmomanometer to monitor blood pressure. The conventional Borg scale (6/20) will be used. **Monitoring:** At the beginning of the session, patients will remain at rest in a seated position for 15 minutes. Heart rate and blood pressure will then be measured. Perceived exertion will be assessed using the conventional Borg scale. This measurement will be repeated during the strengthening and aerobic exercise and at the end of the session. **Intensity:** - Warm-up: To be performed with self-loading activities and position changes. - Central phase: - Strengthening: To be performed at 50% of RM, 3–4 sets for 12–15 repetitions. - Aerobic exercise: To be performed at 50% of HRmax, increasing the intensity every four weeks to a maximum of 70% of HRmax. If on beta-blocker medication, exercise will be prescribed to maintain perceived exertion between 11–13/20 on the Borg scale. - Cool down: To be performed at 50% of HRmax. If on beta-blocker medication, exercise will be prescribed to maintain perceived exertion less than 11/20 on the Borg scale. **Duration:** - Warm-up phase: To be performed for 10 minutes with self-loading activities and position changes. - Central phase: - Muscle strengthening for 20 minutes. - Aerobic exercise for 25 minutes. - Cool down phase for 5 minutes. **Frequency:** Three times a week for 12 weeks. **Professional assistance:** Via Google Meet by a physical therapist specializing in cardiac and pulmonary rehabilitation.
Experimental Group 2: Conventional rehabilitation and Education	**Devices**: This group will perform their intervention face-to-face in the cardiac rehabilitation program. They will use the clinic equipment. **Monitoring:** At the beginning of the session, patients will remain at rest in a seated position for 15 minutes. Heart rate and blood pressure will then be measured. Perceived exertion will be assessed using the conventional Borg scale. This measurement will be repeated during the strengthening and aerobic exercise and at the end of the session. **Intensity:** - Warm-up: To be performed with self-loading activities and position changes. - Central phase: - Strengthening: To be performed at 50% of RM, 3–4 sets for 12–15 repetitions. - Aerobic exercise: To be performed at 50% of HRmax, increasing the intensity every four weeks to a maximum of 70% of HRmax. If on beta-blocker medication, exercise will be prescribed to maintain perceived exertion between 11–13/20 on the Borg scale. - Cool down: To be performed at 50% of HRmax. If on beta-blocker medication, exercise will be prescribed to maintain perceived exertion less than 11/20 on the Borg scale. **Duration:** - Warm-up phase: To be performed for 10 minutes with self-loading activities and position changes. - Central phase: - Muscle strengthening for 20 minutes. - Aerobic exercise for 25 minutes. - Cool down phase for 5 minutes. **Frequency:** Three times a week for 12 weeks. **Professional assistance:** In-person by a physical therapist specializing in cardiac and pulmonary rehabilitation.

HRmax: Maximum Heart Rate

#### Telerehabilitation.

The program will be assisted by virtual technology. It will consist of performing exercises at home for 60 minutes three times a week for 12 weeks. The schedules for the telerehabilitation sessions will be agreed with the patients for their full participation. The exercises will be structured as follows: Warm-up, upper and lower limb muscle strengthening, continuous aerobic exercise, and return to rest. A cardiac and pulmonary rehabilitation physiotherapist will monitor the exercises via the Google Meet platform. This intervention will be carried out by experimental groups 1 and 3.

#### Telephone follow-up.

Telephone calls will be made three times per week (on days other than those assigned for telerehabilitation) as a follow-up method to obtain information from each person on heart rate and perceived exertion during home activities that they will be encouraged to perform outside of the intervention days. This intervention will be carried out by experimental group 1.

#### Conventional outpatient rehabilitation.

The program will be carried out in the cardiac rehabilitation program of the clinic. It will consist of physical exercises supervised by a physiotherapist specialized in cardiac and pulmonary rehabilitation for 60 minutes, three times a week for 12 weeks, structured as follows: warm-up, strengthening of upper and lower limbs, continuous aerobic training, and return to rest. This intervention will be carried out by experimental group 2.

#### Education.

It will be given once a week for 30 minutes to each patient individually and in groups. Topics will cover disease knowledge, medication use, warning signs, anxiety management, relaxation techniques, home exercises, sexual intercourse, and proper nutrition. This component will be delivered in person for experimental group 2 and virtually through the Google Meet platform for experimental groups 1 and 3. The meeting schedule will be arranged with all participants to ensure full availability for connection.

#### Reasons for stopping the intervention.

If, during the intervention, the patient experiences difficulty performing the exercise, symptoms of discomfort, angina, or changes in vital signs that interfere with the continuation of rehabilitation, the patient will be reassessed and withdrawn from the study.

### Data collection, management, and analysis

The information obtained will be recorded in a Microsoft Office Excel 2010 database and exported for statistical analysis to the Statistical Software for Data Science (Stata) V.16. A description of the outcome variables and the population characteristics will be carried out to display the statistical behavior of these variables. Qualitative variables will be presented as frequencies and percentages, while quantitative variables will undergo the Kolmogorov-Smirnov test to determine whether their behavior is parametric or non-parametric.

If parametricity is confirmed, a Levene’s test for equality of variances will be conducted. Once parametricity is established, a paired t-test will be performed to compare pre- and post-intervention results for each group. For non-parametric behavior, the Wilcoxon test will be used, and variables will be presented as medians and interquartile ranges. Additionally, comparisons of the three experimental groups will be made at the start and end of the intervention. If the variables exhibit parametric behavior, a one-way ANOVA test will be used, followed by post hoc tests. For parametric variables with equal variances, the Tukey test will be applied (adjusting for unbalanced groups with Dunnett’s T3 test). If equal variances are not assumed, Dunnett’s T3 test will be used. For non-parametric variables, the Kruskal-Wallis test will be employed. A significance level of 95% will be considered, with a p-value < 0.05 regarded as statistically significant.

An analysis of covariance (ANCOVA) regression model will be used to evaluate the main effect of the outcome variable among the three experimental groups, adjusting for age, sex, and other potential confounding factors (those statistically significant at the 5% level).

A logistic regression model will be considered to analyze a binary outcome (e.g., adherence to recommendations provided through telephone follow-up), utilizing Akaike’s information criterion to select the best model based on its bias and variance.

## Discussion

This protocol study highlights the research design and methodology of a three-arm, blinded, controlled clinical trial to determine the effects of cardiac telerehabilitation, education, and telephone follow-up versus conventional treatment in patients with HF according to the SPIRIT 2023 list guidelines. Therefore, this protocol provides a detailed description of the study to allow replication and comparison with future studies involving this type of population. To date, and according to the literature review performed, this study could be the first clinical trial with three intervention arms comparing telerehabilitation, conventional rehabilitation, education, and telephone follow-up in patients diagnosed with HF.

During both the implementation and the initial phases of the program, several studies have focused on patient education regarding the devices used for delivering and monitoring telerehabilitation [8,19,20]. Other research specifically provided information about HF related to the platforms used in the program [21]. In this instance, educational sessions will cover eight topics that extend beyond HF. These topics align with those presented by Varsamo et al. [22], Hwang et al. [23], and Ewa Piotrowicz et al. [8] in their studies on conventional outpatient rehabilitation and telerehabilitation groups. A consensus has been reached on the importance of an interdisciplinary approach and the necessity of patient health education to ensure treatment efficacy and sustainable outcomes. These educational sessions will be conducted at least once a week.

Van Leunen et al. [19], Varsamo et al. [22], and Xingchen Peng et al. [24] assessed participants for psychological aspects such as anxiety and depression using the HADS and GAD-7 scales. Other authors [25] assessed the quality of life using the SF-36 questionnaire and functional capacity using the Duke Activity Status Index (DASI). In addition, some studies [8,22] assessed biomarkers with laboratory tests and aerobic capacity with the 6MWT in most cases [8,20,22,24,26]. These approaches are comparable to the assessments in this study since, in addition to anthropometric data, aerobic and functional capacity, depression questionnaire, and HRQoL are assessed. Therefore, the similarity of the assessment methods strengthens the consistency and comparability of the results obtained for this study.

Regarding the frequency and duration of the telerehabilitation program, significant differences in the duration of the program in weeks are observed among the different studies, with periods ranging from 8 to 18 weeks, according to the literature on the subject [8,19,20,25,26]. This study defines a 12-week intervention program with sessions scheduled three times per week. This approach is similar to previous clinical trials, which suggest a frequency of intervention between 3 and 5 times per week [8,19,20,22,24]; moreover, it is consistent with the cardiac rehabilitation sessions generally prescribed in Colombia [6].

Concerning exercise prescription, the session is divided into three main phases: warm-up, core training, and cool-down. Concerning intensity, a range of 50% to 70% of HRmax and a value of 11–13/20 according to the conventional Borg scale are established. These results are congruent with those reported in other telerehabilitation studies for patients with HF [8,23–25] and reflect appropriate exercise prescriptions for patients and replication of the study. In addition, to track and monitor the prescription in this study, patients are given an FT4 fleece belt and a digital blood pressure monitor, similar to some studies in which patients were given digital devices to monitor vital signs [9,10,12,17,18] or taught how to take their vital signs [14].

Mobile technology in healthcare allows assessment, measurement, intervention, and remote follow-up synchronously or asynchronously, enabling its application in CR, improving cost-effectiveness, time and interaction with patients, and treatment adherence [27]. Studies involving telephone follow-up have generally been conducted in conventional outpatient rehabilitation programs or at home [28–30], This study aims to provide further insights into cardiac telerehabilitation programs for patients with HF. It demonstrates positive effects on quality of life, reductions in stress and depression levels, and improvements in treatment adherence, thereby facilitating the achievement of therapeutic goals, similar to what was presented in other studies [27,31].

Some limitations of this study include the potential dropout of patients from any of the three groups due to personal or health-related factors, especially in the conventional rehabilitation group, where patients are required to travel to the rehabilitation clinic.

The protocol of this study may present some limitations, such as participant bias due to the sample being drawn from a single clinic, a potential dropout rate, and difficulties with virtual connectivity. However, measures have been proposed to minimize these limitations, including randomization and proper sampling of the participant population, scheduling coordination with participants, and ensuring they have a stable internet connection.

## Supporting information

S1 Checklist SPIRIT 2013 checklist: recommended items to address in a clinical trial protocol and related documents*. (DOCX)
