# Supplementary material for: Study protocol for a controlled clinical trial on cardiac telerehabilitation and educational follow-up in patients with heart failure
Source: PLoS One. 2025 Jul 10;20(7):e0327366. doi: 10.1371/journal.pone.0327366 (PMC12244582; doi:10.1371/journal.pone.0327366)
Supplement: S1 Checklist — (DOCX) [file pone.0327366.s001.docx]

1. **GENERAL PROJECT INFORMATION:** all requested information must be recorded.

| **Project Type:**  Research x | | | | | |
| --- | --- | --- | --- | --- | --- |
| Title: Physical exercise and telephone monitoring mediated by cardiac telerehabilitation in patients with heart failure | | | | | |
| **Date:23 Jun 2022** | | | | | |
| **Principal Investigator:**  **Jhonatan Betancourt Peña**  **Coinvestigador(es):**  **María José Martínez Patiño**  **Iago Portela Pino**  **Juan Carlos Avila Valencia**  **Signature Principal Investigator:** 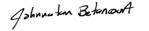 | | | | C.C 1130626652  DNI 32746175V  DNI 35575466D  C.C 16287027 | |
| Email:  **j**honatan.betancourt@endeporte.edu.co | | | | cellphone:  3177602534 | |
| **Name of the Research Group: SEINCAR research hotbed**  **GIESS RESEARCH GROUP**  **Recognized by Colciencias Yes x No ** | | | **Total Researchers (number)**  **4** | | |
| Endorsement of the Research Group to present the project: Yes  Leading firm of the Group: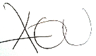 | | | No. of the minutes of the group meeting where the project is endorsed: #04-2022 | | |
| **Participation of another entity in the investigation**  **Yes X No **  **Name: Clínica de Oeste S.A.** | | | Contact details  6603000 EXT 298-172 | | |
| **Line of Research of the group to which it responds:**  Professional development in physiotherapy | | | | | |
| **Descriptors / Keywords (Maximum 5):**  **Exercise, Heart failure, Quality of life, Rehabilitation, Telerehabilitation** | | | | | |
| **Possible Evaluators**  Name, email, telephone number and institution to which you belong of up to 5 expert researchers on the topic of your proposal, who are capable of evaluating projects on this topic. Minimum 3 must not belong to the END UI**:** | | | | | |
| **Name** | **email** | **cellphone** | | | **Institution** |
| **Oscar Ruíz** | **oscar.ruiz91@hotmail.com** | **3206975321** | | | **Universidad María Cano** |
| **Jorge Daza** | **jorge.daza01@usc.edu.co** | **3108923676** | | | **Universidad Santiago de Cali** |
| **Diana Diaz Vidal** | **dianitadv@gmail.com** | **3113042795** | | | **Universidad San Buenaventura** |
| **Esmeria Tapia** | **esmetaca@hotmail.com** | **3235897225** | | | **Universidad de Santander** |
| **Beatríz Muñoz** | **beatriz.munoz@correounivalle.edu.co** | **3172231187** | | | **Escuela Nacional del Deporte** |

**2. SUMMARY/SUMMARY OF THE RESEARCH PROJECT**

**Introduction:** Cardiovascular diseases are the main cause of death and disability worldwide, representing approximately 31% (17.9 million) of total deaths each year. The COVID-19 pandemic has led to the total suspension of most cardiac rehabilitation programs at the highest peaks of the spread, forcing people not to leave home, enhancing metabolic conditions and generating greater complications due to a sedentary lifestyleb. CD. Physical exercise is an essential component in the rehabilitation of patients with heart failure disease, showing improvements in quality of life, functional capacity, in addition to reducing the mortality rate, number of rehospitalizations, and levels of depression,e,f, gram. It is necessary to carry out interventions adapted to the needs of patients who have difficulties traveling to health centers; however, some authors report that remotely oriented exercise could present results similar to those of traditional rehabilitation in a center or clinic, so promoting a cardiac telerehabilitation program together with telephone educational follow-up could cause greater improvements compared to other cardiac rehabilitation and telerehabilitation programsah,i,j.

**Objective:** Determine the effects of a physical exercise program and telephone educational follow-up mediated by cardiac telerehabilitation in patients with heart failure on functional capacity, depression and health-related quality of life.

**Methodology:** A randomized controlled clinical trial type study will be carried out, in a period of 6 months included in the year 2022, in patients with a diagnosis of heart failure who enter a cardiac rehabilitation program in the city of Santiago de Cali, Colombia. to the investigation after the consultation with cardiology in three groups: cardiac telerehabilitation program with physical exercise and conventional educational component with telephone follow-up (Intervention), physical exercise program and conventional educational component mediated by cardiac telerehabilitation and physical exercise program and component conventional educational program to which measurements of sociodemographic variables and before-after clinical measurements (physiological, paraclinical, anthropometric) functional capacity, quality of life and state of depression will be carried out.

**Expected results:** All groups are expected to improve functional capacity and clinical variables, however, the intervention group will present greater improvements in the primary outcome variable and added to this will present significant changes in the state of depression and quality of life related to health.

**3. PROJECT DESCRIPTION**

**3.1. RESEARCH PROBLEM (Maximum 1500 words)**

The World Health Organization (WHO) defines cardiovascular diseases (CVD) as a group of disorders of the heart and blood vessels that include coronary heart disease, cerebrovascular diseases and rheumatic heart disease, which in turn top the list of non-chronic diseases. transmissible and are responsible for the main causes of death nationally and globally^1^.

During the coronavirus disease pandemic, patients with cardiovascular diseases were classified as a high-risk group^2^ with potentially higher mortality if infected, especially heart failure (HF) as it is a prevalent condition affecting about 26 million of patients around the world3. Although confinement represents a solution to prevent viral spread, it jeopardizes regular follow-up visits and prevents direct medical evaluation to detect the progression of heart failure and optimize treatment. Furthermore, changes in lifestyle during quarantine could trigger heart failure decompensation and physical deterioration^2^.

The most common manifestation of heart failure (HF) is deterioration of physical capacity resulting in dyspnea, fatigue during exercise and/or at rest, which, in turn, deteriorates health-related quality of life ( HRQOL), highlighting that the evaluated quality of life of patients is an important indicator for the course and success of a therapeutic process, which allows one to function in daily life despite the disease^4^.

Outpatient CR cardiac rehabilitation is a comprehensive intervention in which patients are offered an individualized program in a facility that may consist of one or more modules or group therapies (i.e., exercise training, education, relaxation therapy, psychoeducational prevention therapy, smoking cessation therapy) and/or individual treatment by a psychologist, dietitian or social worker, since this disease causes physical and psychological deterioration that affects the patient's quality of life. Despite the different programs offered, the participation of patients with HF is low^5^.

Data from the EUROASPIRE IV survey suggest that, in the Netherlands, of 72% of patients with cardiovascular disease who were referred for CR, only 83% ultimately attended a CR program (resulting in a participation rate 60%), as well as the participation rates in patients with chronic heart failure (CHF) or arrhythmias where it is evident that their participation is dramatically lower (approximately 1–30%) since these patients are referred less frequently^5^.

Factors that make CR attendance difficult include lack of resources (lack of caregivers, long waiting lists), as well as logistical and psychological problems (transportation problems, need to be assisted by family members or partners, lack of motivation, not acceptance of the proposed RC models). The development of new technologies provides the opportunity to include telerehabilitation in the organization of HF outpatient care^3^.

One of the strategies used to guarantee continuity of care for patients with HF is telecardiac rehabilitation^6-7^, where patients from home can be monitored through dedicated applications, telephone calls or devices, virtual visits and direct triage where patients with signs or symptoms of decompensated heart failure are evaluated and hospital care can benefit from remote communication platforms, thus allowing these patients to receive remote monitoring or telerehabilitation to avoid early readmissions and reduce associated complications^2^.

Individual health data (e.g., heart rate [HR] during exercise, daily physical activity [PA], or nutritional intake) is monitored to enable personalized feedback and education from a healthcare professional. Recent systematic reviews and meta-analyses show that multidisciplinary telerehabilitation or exercise-based telerehabilitation is a safe and equally cost-effective alternative to conventional center-based CR in patients with heart failure^5^. At the same time, reinforcing the activities carried out in the CR by telephone follow-up is a little explored alternative.

Telerehabilitation can resolve several barriers at the level of the patient, the health professional and the (health care) system that hinder the use of CR and the sustainability of its effects. Examples of these barriers include transportation difficulties (patient level nte), under CR medical support (professional level) or limited facilities to provide supervised exercise training (system level)^5^. It is important to keep in mind that many of the abandonments of conventional rehabilitation are due to the little or no follow-up that is carried out on the patient when they enter the program^8^, so it is essential that rehabilitation programs can generate effective strategies that promote adherence of patients and reinforce the educational component to achieve the intervention goals.

Unfortunately, due to disease-related limitation, not all patients with HF have the opportunity to fully benefit from traditional standard outpatient rehabilitation^4^. Thanks to the implementation of telemedicine systems, cardiac telerehabilitation can be an alternative for these patients^4^. However, little is known about the impact of this new method on the HRQOL and depression status of patients with HF in the Colombian population and even more so with educational reinforcement during rehabilitation sessions.

This is why the following research question is raised: What are the effects of a physical exercise program and telephone educational follow-up mediated by cardiac telerehabilitation in patients with heart failure on functional capacity, depression and health-related quality of life?

**3.2. JUSTIFICATION (Maximum 1500 words)**

In the early months of the COVID-19 pandemic, the impact of cardiovascular comorbidities on disease course became evident in observational studies, indicating that patients with prior cardiovascular disease had higher disease severity and mortality. . by COVID-19^6^. Furthermore, myocardial injury has been widely described in COVID-19, which could further deteriorate myocardial function and worsen the prognosis in patients with known HF^2^. For these reasons, the great challenge for patients with HF during COVID-19 is to keep them safe from the risk of infection, but also to continue with strict follow-up to avoid hospitalizations.

Taking into account the aforementioned and knowing that CVD is one of the main causes of death worldwide^1^, behavioral change, modification of risk factors, nutritional counseling, stimulation of psychosocial well-being and the practice of Physical activity is necessary to improve the quality of life of patients with HF^9^. Thus allowing benefits to be generated in the long term with high adherence and achieving a persistent change in lifestyle.

According to the European Society of Cardiology (ESC) guidelines^10^, cardiac rehabilitation is a recognized form of therapy for all patients with stable heart failure. Cardiac rehabilitation improves functional capacity, autonomic function, peripheral hemodynamics and, in most cases, has a beneficial effect on the HRQOL of patients with HF, that is, an increase in the ability to function physically, mentally . and socially^4^.

In most cases, regular and well-planned physical training improves HRQoL, your psychological and mental condition; They become more self-sufficient and independent in the performance of daily activities, which consequently produces the feeling that the illness and disability are affecting them much less than before^4^.

Exercise-based rehabilitation has become a safe and effective intervention for patients with chronic heart failure and is now recommended as standard practice^11-12^. Specifically, exercise-based rehabilitation increases physical function, improves HRQoL, and reduces hospitalization rates^9^. Despite this, participation in rehabilitation remains low. Reported barriers to participation include transportation difficulties, financial cost, embarrassment about participation, and program availability^13^. Cardiac telerehabilitation has been proposed as a complement to cardiac rehabilitation or as an alternative approach that could alleviate some of these barriers^13-15^.

Cardiac telerehabilitation offers a novel model of organization and implementation of the comprehensive management of patients with HF. This multidisciplinary management approach with ICT seems to be a better solution to reduce rehospitalization^16^. Some authors have reported on rehabilitation in a home program for patients with HF demonstrating that the use of technology was safe and feasible, and led to an improvement in clinical physical parameters and quality of life^16^.

Telerehabilitation represents a useful tool to prevent the direct and indirect negative consequences of SARS-CoV-2, and the current situation could be the right time to implement a structured telerehabilitation program in clinical practice, providing optimal treatment to patients. patients with HF^3^, the monitoring of early signs of heart failure of new onset or decline, and reduction of unnecessary visits to the hospital to reduce the incidence of group infections^3^, all of this articulated with an adequate educational strategy that allows maintaining healthy behaviors in the long term . .

The social and environmental effects of confinement should also be mentioned, which will greatly affect this population, leading to a change in their lifestyle, changes in diet, increased alcohol consumption and decreased physical activity, which They are important factors to take into account since they can trigger HF^3^ decompensations, so they must be contrasted through educational strategies focused on maintaining adherence to healthy behaviors in the population under study.

For these reasons, the great challenge for patients with HF during COVID-19 is to keep them safe from the risk of infection, but also to continue with strict follow-up to avoid hospitalizations^17^. In this context, various telerehabilitation and remote monitoring strategies such as telephone follow-up were rapidly developed and more widely implemented in patients with HF^3^. Even the strengthening Educational component, both in-person and mediated by technologies, could generate greater benefits in cardiac rehabilitation programs in order to maintain intervention goals in the medium and long term.

The benefits of cardiac rehabilitation in patients with heart failure (HF) are well established. Therefore, current guidelines strongly recommend physical training as an important component of HF management, in addition to linking educational reinforcement activities^4^, and with home-based telerehabilitation it will help overcome these barriers and increase rehabilitation adherence. cardiac due to its convenience and accessibility, having positive effects on the health outcomes of patients with HF^18-19^.

**3.3. STATE OF THE ART (Maximum 1500 words)**

Coronavirus disease 2019 (COVID-19) infection, caused by severe acute respiratory syndrome, poses a dual threat to public health: the immediate threat is infection-related morbidity and mortality, and the more subtle threat is It is the change of attention and resources away from the care of common diseases^20^.

Studies show that during the pandemic, a paradoxical reduction in hospitalization rates for heart failure was observed, caused by patients' reluctance to go to emergency rooms and hospitals, which could result in higher patient mortality and/or hospital admissions. more complicated heart failure in the future^21^.

The literature confirms that COVID-19 represents a serious threat to the heart failure population due to both the increased risk of severe illness and death and the reduced availability of outpatient care^22^.

Studies show that telerehabilitation can be adopted in all its different forms and possibilities to guarantee the continuous provision of medical care to patients with heart failure^2^, even adopting monitoring strategies such as telephone monitoring, SMS text messages or WhatsApp. The term telerehabilitation has been used in much of the literature to date and is defined as the provision of rehabilitation services through information and communication technologies^2^. In the face of COVID-19, it has proven to be a viable and effective alternative for people who cannot access in-person health services for the management of many conditions. During COVID-19, the reallocation of medical resources as well as the lockdown led to the cessation of all non-urgent medical services, including CR, therefore, centers had to shift to alternative ways of delivering the core components of CR. Remotely^2,3^.

Studies demonstrate the different CR proposals driven by technology, with the help of any technological form (e.g., smartphones, mobile applications, Internet, email, web cameras and use of wearable sensors)^2^, added to this the reinforcements Through emails, telephone follow-up or WhatsApp messages, they are a cost-effective alternative to CR programs. A recent survey on the implementation of cardiac telerehabilitation services during the COVID-19 pandemic in Belgium showed that half of the response centers switched to telerehabilitation during the pandemic, mainly for patients who were already in CR. The most frequently used medium for delivering CR components was online videos (71%), followed by website information (64%) and emails (64%). As the authors of this survey suggested, remote delivery of CR may also play an important role following the reopening of rehabilitation centers due to reduced capacity and social distancing measures^2^.

Exercise is an essential component within cardiac rehabilitation, patients with HF generate benefits, as evidenced in the studies reported by Jolly et al. and Oka et al. Where patients in home rehabilitation improved in the mental and physical component. The authors highlighted the importance of support from medical staff who monitor the progress of home rehabilitation. They also noted the role that feedback information played in home rehabilitation^4^. As in the study by Quittan et al. where they reported that the improvement indicator mainly referred to the general feeling of health, as well as physical and social functioning. The authors pointed out the importance of reducing dyspnea and fatigue, which was reflected in better quality of life results^4^. And finally in the study by Koukouvou et al. indicated an equally positive effect of cardiac rehabilitation in the form of increased physical fitness and improvement in general health status in patients with HF^4^. Other studies confirm that patients with HF benefit from physical training and psychological assistance, which they receive with the parallel support of the rehabilitation team (doctor, physiotherapist, nurse) since during confinement both their mental and physical health is affected, generating various decompensations of IC^4^.

Studies provide evidence that it is feasible, safe, effective and capable of maintaining results for 6 months, in the elderly population with chronic and combined medium-severe heart diseases who are in stable clinical condition after a period of hospital rehabilitation^8^.

The feasibility and safety of the telerehabilitation program is evidenced in a study where an effectiveness is observed ad significant in exercise tolerance (6MWT), after 4 months, where patients walked an average of 60 m more compared to baseline. Likewise, patients reduced dyspnea and maintained a better physical activity profile with decreasing impairment/disability, better QoL, and more time free of new events (all-cause readmissions and death)^8^.

A hybrid trial of comprehensive telerehabilitation in patients with HF (TELEREH-HF) was designed to determine whether potential improvements in functional and quality of life outcomes after a 9-week training period translate into improved outcomes. clinical outcomes during extended follow-up of 12 to 24 months compared to usual care (UC)^2^ where high prognostic factor adjustment was achieved, exercise training was associated with modestly significant reductions for both all-cause and hospitalization as well as for mortality due to CVD or hospitalization due to HF^2^

Telehealth rehabilitation without regular clinic and home visits has had positive effects on the health outcomes of patients with HF. Clark et al conducted a meta-analysis that included^14^ randomized controlled trials of remote monitoring (structured telephone support or telemonitoring) to investigate its effects in patients with heart failure and found a decrease in heart failure admissions, a reduction in mortality for all the causes, a better quality of life, and reduced health costs. Piotrowicz et al demonstrated that both home-based telehealth cardiac rehabilitation and standard clinic-based cardiac rehabilitation significantly improved quality of life, but home-based telehealth cardiac rehabilitation showed better adherence compared to standard cardiac rehabilitation^4^.

Exercise-based rehabilitation has become a safe and effective intervention for patients with chronic heart failure and is now recommended as standard practice. Specifically, exercise-based rehabilitation increases physical function, improves quality of life, and reduces hospitalization rates^5^.

Therefore, it is necessary to implement a physical exercise program through telerehabilitation to determine the benefits that are generated in patients to improve their functional capacity, clinical variables, depression and health-related quality of life, which allow an impact and change. positive in your lifestyle.

**3.4. THEORETICAL FRAMEWORK**

For the comprehensive approach to this research, it is necessary to define key concepts, which include:

**Cardiac Rehabilitation**

According to the WHO, cardiac rehabilitation is “the set of activities necessary to ensure people with cardiovascular diseases an optimal physical, mental and social condition that allows them to occupy, by their own means, a place as normal as possible in society.” society”^23^.

Evidence has shown that formal exercise or any form of physical activity is associated with a marked reduction in mortality in individuals with and without coronary heart disease. In a study conducted in Olmsted, Minnesota, patients who attended rehabilitation programs reduced cardiovascular events by 25% for each increase of one metabolic equivalent (METS) in exercise capacity^24^.

**Components of Cardiac Rehabilitation**

According to the consensus statement of the American Heart Association, the American Association of Cardiopulmonary Rehabilitation and the American College of Cardiology (AHA/AACVPR/ACC)^25^, a cardiac rehabilitation program includes the comprehensive care of patients with CVD and chronic heart failure. (class I evidence). Rehabilitation should include a multidisciplinary approach consisting not only of exercises, but also concentrating its efforts on educational work in order to achieve control of risk factors through lifestyle modification and adherence to healthy lifestyle habits^24^.

**Cardiac Telerehabilitation**

It is that intervention in which rehabilitation treatment is provided to patients remotely based on the application of information and communication technologies (ICTs)^26^. It has been shown that patients with cardiovascular diseases show great interest in technologies, favoring the telemonitoring process during their rehabilitation process. In this way, supervision and control of the patient is guaranteed throughout the intervention^26^.

Taking into account the concepts mentioned above, it is necessary to define a model that addresses them comprehensively, taking into account these key aspects, the biopsychosocial model proposed in 1977 by Engel^27^ is proposed.

The biopsychosocial model states that a simple biochemical alteration is not enough to explain all the ailments of individuals; For this reason, it is important to integrate psychosocial variables when evaluating the patient since they determine the susceptibility, severity and course of the condition that a person could have^27^.

This model constitutes an option to promote the holistic approach of comprehensive care for people with HF and transcends the biomedical approach by emphasizing the interaction of the person and the environment that influences and conditions the disease. According to Borrell^16^, this model promotes a more comprehensive knowledge of the patient and his environment, integrates the findings in the three spheres (biological, psychological and social) Figure 1, recognizes the central role that the therapeutic relationship plays in the course of events, optimizes Teamwork incorporates the health professional as another piece of the system that must also be cared for^27^.

The biopsychosocial model in clinical practice could favor the evaluation of patients through a comprehensive approach from primary health care. This could guide an improvement in the quality of care for patients from the primary level and influence the reduction of preventable hospitalizations due to HF through better control of the pathology^28^.

In the Colombian health context, this could favor the well-being of users, impact health outcomes by reducing preventable hospitalizations due to HF and thus reduce care costs from primary care^28^; The evaluation of biological, psychological and social factors helps the health professional to carry out a comprehensive assessment during the time of care in health institutions.

However, the biopsychosocial model extends beyond medical care and examines the patient's biological, psychological, social, comorbidities, illness beliefs, coping strategies, fear, depression, employment, and unique concerns and may provide greater understanding. which has hindered recovery^28^. Given this paradigm, a qualified physiotherapist, in contact with the patient, could determine the benefits that are generated in patients to improve their health-related quality of life through a cardiac telerehabilitation exercise program with a conventional educational component and telephone follow-up.


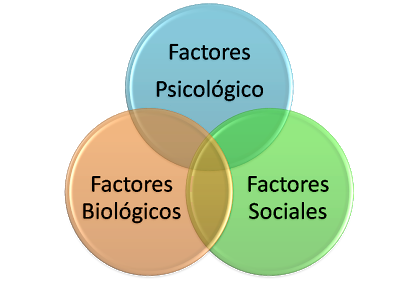
Figure 1. Biopsychosocial Model

**3.5. LEGAL FRAMEWORK**

Regarding the CR, in the legislation, the Colombian government under resolution 4252 of 1997 which establishes the technical, scientific and administrative standards that contain the essential requirements for the provision of health services, and in turn establishes the registration procedure. thereof. This decree defines that in terms of human resources, cardiac and pulmonary rehabilitation programs must have professionals trained in this area either through specialization-type postgraduate education, or have well-founded experience in the area. Likewise, through ruling T-760 of 2008, the National Council of Social Security in Health in its first article which talks about the contents of the mandatory health plan, touching on the issue of health recovery actions in the high-cost diseases such as CD, guaranteeing that activities, interventions and procedures framed in physical medicine and rehabilitation are provided and specifically offering CR programs for cases contemplated surgical or not, in order to provide rigorous medical control and a adequate treatment in the Republic of Colombia.

Law 528 allows physiotherapy professionals to carry out technical-scientific activities related to research. Being a self-employed professional, the framed activities will be carried out with what is permitted by law and the provisions framed by the Ministry of Health.

**3.6. OBJECTIVES (Maximum 500 words)**

**GENERAL OBJECTIVE**.

To determine the effects of a physical exercise program and telephone educational follow-up mediated by cardiac telerehabilitation in patients with heart failure on functional capacity, depression and health-related quality of life.

**SPECIFIC OBJECTIVES.**

- Sociodemographically and clinically characterize the study population
- Determine the effects of a physical exercise program and conventional educational component in patients with heart failure on functional capacity, depression, and health-related quality of life.
- Determine the effects of a physical exercise program and conventional educational component mediated by cardiac telerehabilitation in patients with heart failure on functional capacity, depression and health-related quality of life
- Determine the effects of a physical exercise program and educational component with telephone follow-up mediated by cardiac telerehabilitation in patients with heart failure on functional capacity, depression and health-related quality of life
- Estimate differences in functional capacity, depression, and health-related quality of life between the intervention group and comparison groups.

**3.7. PROPOSED METHODOLOGY (Maximum 2500 words)**

**Type of study**

Controlled clinical trial type study, in patients entering a cardiac rehabilitation program during the year 2022.

Linking to the research after the consultation with cardiology in three groups: physical exercise program and conventional educational component with telephone follow-up (Intervention), physical exercise program and conventional educational component mediated by cardiac telerehabilitation, physical exercise program and conventional educational component to which measurements of sociodemographic variables and before-after clinical measurements (physiological, paraclinical, anthropometric) functional capacity, depression and health-related quality of life will be carried out.

**Null hypothesis**

• There are no differences between a physical exercise program and educational component with telephone follow-up mediated by cardiac telehabilitation in patients with heart failure in functional capacity, depression and health-related quality of life compared to groups that do not receive it.

**Alternate hypothesis**

• There are differences between a physical exercise program and educational component with telephone follow-up mediated by cardiac telehabilitation in patients with heart failure in functional capacity, depression and health-related quality of life compared to groups that do not receive it.

**Population and sample**

The population is represented by patients with a diagnosis of heart failure who enter a cardiac rehabilitation program in the city of Santiago de Cali, Colombia; It is expected to evaluate patients during a period of 6 months in 2022.

It should be taken into account that this study aims to compare quantitative variables:

Studies that compare means use the standard deviation as a measure of variation. The aim is to know if the means are different, higher or lower in one group than in the other and it is necessary to have the following data:

1. Determine the expected measurement of the variable of interest in both groups.

2. Identify if the hypothesis is unilateral or bilateral.

3. Define the level of confidence desired. Generally, we work at 95%, which implies a probability of error α = 5% (0.05), but it can be higher (99%, error α = 0.001 or 1%) or lower (90%, error α = 0.1 or 10%). ) according to what you are looking for.

4. Establish the power of the study (1 – β). It is generally set to 80%, which means accepting the probability of a β error of 20%. Remember that a β error of 20% means an 80% probability of detecting a difference if it exists in reality.

5. With the level of confidence and the proposed power of the study, consult in table 8 (taken from the book Sampling and sample size, a practical guide for health personnel who carry out research) the values ​​of Zα and Zβ added and squared, which constitute the constant K of the formula.


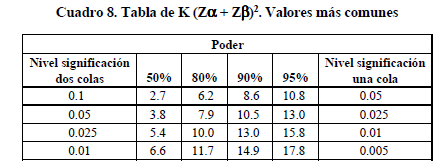


6. Determine the difference in means that we would expect to find or that we want to be able to detect with our study (μ1 – μ2 – μ3).

7. Determine the expected standard deviation in each group.

8. Substitute into the formula:

Sample calculation:

Group 1: conventional

Group 2: telerehabilitation

Group 3: telerehabilitation and telephone follow-up

minutes.

H0: vs μ2

H0: μ1vs μ3

H0: vs μ3

Ha: μ1- μ2 ≠ μ3

μ3: average of the distance traveled in the 6-minute walk test of group 3 (estimated from clinical experience, taking group 2 as a reference since there are no studies with similar characteristics with telephone follow-up, so it would be obtained a distance traveled equal to the telerehabilitation group as the groups are homogeneous at the beginning of the intervention but heterogeneous at the end of the intervention program)

Confidence: 95%

α error: 5% (0.05)

β error: 20% (0.20)

Power: 1-β: 80%

K: 7.9 (value taken from the table K(Zα+Zβ)2 )

Comparison of two independent means (STATA version 14)


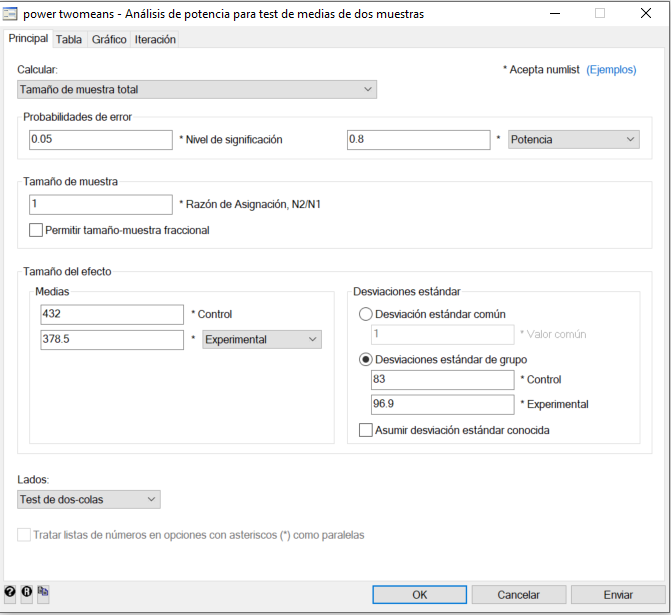


n= 46 participants per group. It increases by 12% due to possible problems in measurements or abandonment of study participants, such that n = 52 patients per group are required.

Total number of subjects for the 3 groups: 156

The randomization of patients into the intervention groups will be carried out once they complete the consultation with a specialist doctor and sign the informed consent for the study, who will verify the inclusion criteria to enter the CR program.

The patients will then be referred to an external person who, with the Microsoft Excel program, will proceed to number the boxes in a table to which a simple randomization formula will be applied, in this way the patients will be registered in the database in the numerical order that are linked to the study and the group to which the patient would belong will automatically be assigned according to randomization.

The result will be sealed in a manila envelope and delivered to an educator trained in telephone follow-up (in the intervention group) who will not be part of the work team, who will be in charge of carrying out the educational telephone follow-ups to the patients randomly in the study.

Later, the schedule and CR sessions will be assigned to them. It is expected to have double blinding, by the principal investigator and the health professionals participating in the CR program.

Patients will be recruited at the rehabilitation center; where the research will be explained in detail and any doubts will be resolved to subsequently sign the informed consent (ANNEX 1), if the following inclusion criteria are met.

**Inclusion criteria:**

- Patients previously diagnosed by a Cardiology Specialist with heart failure.
- Patients who have an indication to exercise.
- Patients who have digital equipment such as a computer, tablet or cell phone with access to virtual platforms to carry out exercise sessions mediated by technology.
- Participants who accept and sign the informed consent.

**Exclusion criteria**

- patients with respiratory comorbidity or who present some limitation in performing active and resisted movements (recent fractures, recent hemodynamic alterations, coronary heart disease event after diagnosis of cardiovascular disease, infectious diseases and neuromuscular limitation).

**Operational Definition of Variables**

**Operational table of Sociodemographic variables**

| **VARIABLES** | **CATEGORIES** | **OPERATIONAL DEFINITION** | **INSTRUMEN** |
| --- | --- | --- | --- |
| Gender | Nominal qualitative | sexual identity of living beings | Entry format |
| Age | Quantitative ratio | Time elapsed since the birth of the subject |  |
| Home | Nominal qualitative | geographical area composed of different municipalities |  |
| City |  |  |  |
| Stratum | Ordinal qualitative | group of people within a society separated by socioeconomic level |  |
| Education level | Ordinal qualitative | The subject's level of education is established through the initial interview. |  |
| Occupation | Ordinal qualitative | which spends most of the time paid or unpaid |  |
| Civil status | Nominal qualitative | set of legal situations from which people relate to their family of origin, or that they have formed, and with elements of legal personality that are fundamental in themselves |  |
| EPS - health regime | Nominal qualitative | Character of the health service provider entity that refers the patient to the pulmonary rehabilitation program |  |

**Condición de ingreso**

| **VARIABLES** | **CATEGORIES** | **OPERATIONAL DEFINITION** | **INSTRUMEN** |
| --- | --- | --- | --- |
| Diagnosis | Nominal qualitative | It is the diagnosis given by the referring doctor and with indication of pulmonary rehabilitation. | Medical history - entry form |
| Comorbidities/other DX | Polytomous Categorical | Underlying pathological condition that worsens the prognosis of the disease |  |
| Visits to the emergency room last year | Quantitative ratio | Number of visits to the emergency room due to respiratory illness in the last year |  |
| Hospitalizations last year | Quantitative ratio | Number of hospitalizations due to respiratory illness in the last year |  |
| Type of hospitalization | Ordinal qualitative | It refers to the type of hospitalization according to clinical condition and that the stay is longer than one day. | Medical history - entry form |
| Medicines | Nominal polytomous qualitative. | Determines the drugs that are administered to the patient as part of their medical treatment |  |
| Exposure to wood smoke | Ordinal qualitative | years spent exposed to tobacco smoke |  |
| Smoke | Qualitative polytomous nominal | action indicating tobacco consumption |  |
| Home oxygen | Ordinal qualitative | use of therapeutic action that consists of the administration of oxygen at concentrations greater than those found in ambient air. |  |
| Physical activity level | Ordinal qualitative | It is identified as the physical condition and energy expenditure derived from the physical activity that the patient has before and after performing |  |
| NYHA Classification | Ordinal qualitative | a pulmonary and cardiac rehabilitation program Order in functional severity of the disease in numbers  1. NYHA I  2. NYHA II  3. NYHA III  4. NYHA IV |  |

**Antropométricas**

| **VARIABLES** | **CATEGORIES** | **OPERATIONAL DEFINITION** | **INSTRUMEN** |
| --- | --- | --- | --- |
| Size | Continuous quantitative | Patient height in meters | Medical history - entry form |
| Weight | Continuous quantitative | Measurement of the gravitational force on a body expressed in kg |  |
| Abdominal perimeter | Quantitative ratio | Circumference of the abdomen taken from bony prominences |  |
| BMI | Ordinal qualitative | Measure of association between the patient's weight and height is defined as BMI=WEIGHT KG/HEIGHT MT2 |  |
| BMI | Quantitative ratio | Measure of association between the patient's weight and height is defined as BMI=WEIGHT KG/HEIGHT MT2 |  |
| IAC | Ordinal qualitative | Measurement resulting from the relationship between height divided by abdominal perimeter |  |

**Dyspnea and aerobic capacity**

| **VARIABLES** | **CATEGORIES** | **OPERATIONAL DEFINITION** | **INSTRUMEN** |
| --- | --- | --- | --- |
| Modified Borg scale | Ordinal qualitative | Describes the patient's perception of drowning | Medical history - entry form |
| Scale perception of fatigue effort in lower limbs | Ordinal qualitative | Describes the patient's perception of fatigue |  |
| Distance traveled test walk 6 minutes | Continuous quantitative | It is the distance traveled in a non-incremental submaximal test lasting 6 minutes measured in meters. |  |
| **Predicted distance** | Continuous quantitative | It is the estimated distance to travel for the patient according to Enright.  MEN  DISTANCE 6 MINUTES (m)=  (7.57 x height cm) – (5.02 x age) – (1.76 x weight Kg) -309 m  WOMEN  DISTANCE 6 MINUTES (m)=  (2.11 x height cm) – (2.29 x weight Kg) – (5.78 x age) + 667 m |  |
| Percentage of distance traveled 6-minute walk test | Qualitative reason | Estimated percentage covered in the 6-minute walk test in relation to the predicted distance  It is measured in percentage: distance traveled X 100/predicted distance | HC- entry form |
| Estimated Vo2 | Qualitative reason | It is the estimated oxygen consumption necessary to cover a distance in the 6-minute walk test.  Estimated VO2 = Speed ​​(m/min) x 0.1 + 3.5 mLO2/Kg/min. |  |
| Estimated Mets | Qualitative reason | It is the unit of metabolic equivalent necessary to travel a distance in meters in the 6-minute walk test, measured in METS = VO2/3.5 |  |
| Heart rate | Continuous quantitative | Number of beats the heart makes in one minute, measured in beats/minute. |  |
| Breathing frequency | Continuous quantitative | Number of breaths in one minute is measured in breaths/minutes |  |
| Blood pressure | Continuous quantitative | Pressure level exerted by blood within the arteries measured in mmHg |  |
| **Arterial oxygen saturation** | Continuous quantitative | Saturation of oxyhemoglobin with oxygen in arterial blood measured in percentage |  |

**Variable Depresión**

| **VARIABLES** | **CATEGORIES** | **OPERATIONAL DEFINITION** | **INSTRUMEN** |
| --- | --- | --- | --- |
| Depression domain | Continuous quantitative | Determine the relationship between your illness and depression | **PHQ-9 Questionnaire** |

**Variables Health-related quality of life**

| **VARIABLES** | **CATEGORIES** | **OPERATIONAL DEFINITION** | **INSTRUMEN** |
| --- | --- | --- | --- |
| Quality of life | Continuous quantitative | Determines the individual's perception of their health status in relation to their illness. | **MLFHQ Questionnaire** |

**Collection and Sources of Information**

The present study will have primary and secondary sources of information. For the second case, information will be obtained from the patient's medical history through the RIPS and referral by a specialist doctor. Data will be extracted from medical history such as: medications, hospitalizations in the last year, visits to the emergency room, comorbidities, main diagnosis and diagnostic tests.

Regarding the sources of primary information, participants will be summoned to a first face-to-face session at the clinic to perform an initial physical evaluation. During the initial visit, a physiotherapist specializing in cardiac and pulmonary rehabilitation will explain to the patient the purpose of the study, subsequently. An admission questionnaire will be applied (ANNEX 2) related to sociodemographic conditions, cardiovascular risk factors, symptoms for risk stratification according to the recommendations of the American Heart Association and anthropometric data will be collected, the walking test of the 6 minutes, depression and health-related quality of life questionnaire that will be taken again after 12 weeks of intervention in the CR program.

It is important to mention that a portable 3-lead electrocardiogram monitor will be used, which was obtained through the clinic (this is previously calibrated by the biomedical team) and will be used during the first 4 weeks to monitor vital signs and rhythm. cardiac this in order to provide safety to patients while performing the exercises.

Operational phases of methodological design

**Phase I: Literature review**

A search was carried out for articles in different databases such as OVID, Elsevier, Pubmed, Dialnet, Google Scholar, among others, where files were filtered by keywords in Mesh or Desh terms such as: Quality of Life, Exercise Tolerance, Anxiety, Depression, Telerehabilitation, Heart Failure.

**Phase II: Selection, design, testing and adjustment of instruments.**

A pilot test will be carried out with a detailed survey of 15 patients where the following variables will be included as part of the sociodemographic conditions: gender, age, weight, height, BMI, EPS, Main diagnosis, other diagnoses, drugs being taken, marital status , smoking habit, pack index – year, exposure to wood smoke – year, emergency room visits in the last year, hospitalizations in the last year, days hospitalized in the last year. (ANNEX 2), In turn, it will be confirmed that the measurements of functional capacity, depression and HRQoL are carried out in accordance with the clinic's institutional guidelines.

**Phase III: Linking the population, request for informed consent**

The population entering the cardiac rehabilitation program will be referred by a specialist doctor, the principal investigator will contact potential participants who meet the inclusion criteria and they will be explained in detail what the research consists of. Those who agree to participate will undergo the initial evaluation. Those participants who do not complete the program, but carry out the initial assessments, will receive a telephone call to determine the cause of dropout and the data will be recorded as part of the sociodemographic description of the population under study.

Complete privacy will be ensured in the handling of data obtained during the evaluations. Once the patients agree to be part of the research, informed consent will be given for reading and subsequent signing. (APPENDIX 1). It must be taken into account that the responsibility of the participant begins from the moment they sign the informed consent, in turn, this culminates when the patient voluntarily decides so or when the final evaluation is completed.

The randomization of patients into the intervention groups will be carried out once they complete the consultation with a specialist doctor and sign the informed consent for the study, who will verify the inclusion criteria to enter the CR program.

The patients will then be referred to an external person who, with the Microsoft Excel program, will proceed to number the boxes in a table to which a simple randomization formula will be applied, in this way the patients will be registered in the database in the numerical order that are linked to the study and the group to which the patient would belong will automatically be assigned according to randomization.

The result will be sealed in a manila envelope and delivered to an educator trained in telephone follow-up who will not be part of the work team, who will be in charge of carrying out the educational telephone follow-ups to the patients randomly in the study.

Later, the schedule and CR sessions will be assigned to them. Be esp was to have double blinding, by the principal investigator and the health professionals who participated in the CR program.

It is important to keep in mind that through randomization the participants will be selected into three groups:

Intervention: Physical exercise program and conventional educational component with telephone follow-up (ANNEX 3)

Control 1: Physical exercise program and conventional educational component mediated by cardiac telerehabilitation (ANNEX 3)

Control 2: Physical exercise program and conventional educational component (ANNEX 3)

**Bias control**

| **Bias** | **Control** |
| --- | --- |
| **Selection bias** | Random selection where the researcher does not participate |
| **Information biases** | Data from testing measurements of functional capacity, depression, and health-related quality of life will be collected by the cardiac rehabilitation center and provided to the investigator.  The paraclinical results and other tests will be performed by the same laboratory or professional at the beginning and at the end of the study and their results will be delivered to the researcher.  Personal data will be taken directly from the study participants. The applicability of the surveys will be carried out by a person who is unaware of the interest of the researchers. |
| **Observer biases** | The application of the clinical survey will be applied by a physiotherapist other than the researcher in order to collect the subjects' initial clinical data.  The monitoring and application of the monitoring strategy will be carried out by a nursing assistant who has no personal interests in the study.  The monitoring chart and the collection of information will be carried out by a nursing assistant. |
| **Measurement biases** | The paraclinical tests will be carried out at the beginning and at the end of the study with the same clinical laboratory that has quality certification, which objectively guarantees the quality and calibration of the equipment. Like tests such as the echocardiogram, it will be performed by the same medical staff and calibrated equipment at the beginning and end of the program. |

**Phase IV: Information collection**

For collection, formats and instruments designed to obtain all the data are used. (APPENDIX 2)

Admission diagnosis: Obtained from the interview with the specialist doctor and corroborated by reviewing the patient's medical history.

Other diagnoses: Obtained from the interview with the specialist doctor and corroborated by reviewing the patient's medical history.

Medications: Obtained from the interview with the specialist doctor and corroborated by reviewing the patient's medical history.

Diagnostic tests: Obtained from the interview with the specialist doctor and corroborated by reviewing the patient's medical history.

Smoke: Obtained from the interview with the patient and corroborated by reviewing the patient's medical history.

Current smoker: Obtained from the interview with the patient and corroborated by reviewing the patient's medical history.

Package year number: Obtained from the interview with the patient and corroborated by reviewing the patient's medical history.

Wood smoke exposure: Obtained from the interview with the patient and corroborated by reviewing the patient's medical history.

Exposure time to wood smoke: Obtained from the interview with the patient and corroborated by reviewing the patient's medical history.

Physical activity level is evaluated before and after intervention in the program:

Emergency visits in the last year: Obtained from the interview with the patient and corroborated by reviewing the patient's medical history.

Hospitalizations in the last year: Obtained from the interview with the patient and corroborated by reviewing the patient's medical history.

Days hospitalized: Obtained from the interview with the patient and corroborated by reviewing the patient's medical history.

Health professional who refers to the program: Obtained from the interview with the specialist doctor and corroborated by the review of the patient's medical history.

Gender: Obtained from the interview with the patient

Age: Obtained from the interview with the patient and corroborated by reviewing the patient's medical history.

EPS: Obtained from the interview with the patient and corroborated by reviewing the patient's medical history.

Stratum: It is asked of the patient and is noted on the admission form

Marital status: This is asked of the patient and is noted on the admission form.

City of residence: This is asked of the patient and is noted on the admission form.

Weight: It is taken on a scale and the value in kg is noted on the entry form

Height: It is taken using a stadiometer and the value in cm is noted on the entry form.

BMI: Measure of association that relates WEIGHT in KG / HEIGHT in mt2.

Body adiposity index: taken from abdominal perimeter and height, in the entry form.

In the cardiac rehabilitation unit, those participants who enter a 36-session CR program will be taken into account; whether or not they meet the inclusion criteria will be reviewed; those subjects who meet these criteria will be presented with the research project. where they will be explained what its objective is and the benefits that can be obtained.

Subjects who agree to enter the study will be asked to sign informed consent, they will be given an educational talk about the importance of nutrition and exercise as a modifiable factor in cardiovascular risk, and a clinical survey will be completed where basic information will be obtained to provide information. At the beginning of the study, said survey will be tabulated in Excel 2013. This survey will take into account personal identification data, location, anthropometric measurements and habits related to cardiovascular risk and the result of the aerobic capacity test represented in MET.

Paraclinical data will be collected from the HC of the institution where the cardiac rehabilitation unit is located. The results of said data will be delivered directly to the researcher who will record the results in Excel.

Random selection of the intervention group and the two control groups will be carried out.

The members of the intervention group will be given to the person in charge of carrying out the telephone follow-up with the location data (address, landline and cell phone numbers, and emails) to start the follow-up.

**The monitoring plan will be carried out as follows:**

**• Intervention group.**

You will be offered an educational talk about the importance of nutrition, medication and exercise as an important strategy to change cardiovascular risk factors at the beginning of the study.

They will be called three times weekly, where they are reminded of the importance of performing the exercise and the frequency in which they should do it, with precise notes that avoid confusion such as:

Start of tracking 1: “Remember to do your physical exercise for at least 30 minutes a day 3 or more times a week.”

Start of follow-up 2: “Remember the time at which you should usually do your exercises.”

Start of follow-up 3: “Remember that you must warm up, strengthen your muscles, and predominantly continue aerobic exercise to maintain the benefits of your cardiac rehabilitation exercises.”

Based on the previous statements, the information will be expanded and all doubts related to the exercise will be resolved.

It will be recorded in an Excel table each time said text is sent to the patient indicating that it is an activity carried out, this in order to monitor that said text was actually sent.

They will be sent, at the beginning and on a monthly basis, a brochure explaining how to carry out physical activity at home in case they cannot go for a walk, go to a park or cannot attend a gym.

A survey will be administered monthly where adherence to exercise is measured during the previous month. The survey will rate items such as adherence factors associated with the patient, the environment where the patient lives, and the exercise plan.

These activities will be carried out for 12 weeks. At the end of week 12, patients will be summoned to the cardiac rehabilitation unit in order to take anthropometric data again, the 6-minute walk test, depression questionnaire and health-related quality of life. These results, along with those reported in the clinical history, will be entered into the Excel registration table.

**• Control groups**.

The control groups will not receive telephone follow-up, but at the end of week 12, the two patient groups in the control group will be summoned to the cardiac rehabilitation unit in order to take the anthropometric data again, the 6-minute walk test. , depression and health-related quality of life questionnaire, these results will be delivered directly to the principal investigator and will be entered into the Excel recording table.

Regardless of the intervention or control group, a physical and digitalized file will be kept with the results of the tests where the confidentiality, preservation and custody of said documents will be guaranteed as required by resolution 1995 of 1999 management of the file or clinical history.

**Phase V: Processing or treatment of information**

To process the information, an Excel database is created to which only the main researcher has access, according to the results obtained in the evaluated categories of the survey. This allows information not to be lost and to be presented accurately. orderly manner when tabulating the results in the SPSS 24 statistical package.

**Phase VI: Analysis of the results**

A description of the outcome variables and the characteristics of the population will be made, in such a way that a statistical description of their behavior is shown. Measures of central tendency and measures of dispersion will be taken into account.

The data will be subjected to statistical analysis to determine whether the data are parametric or non-parametric, applying the Kolmogorov-Smirnov test. If it is determined that there is parametricity in the data, a statistical analysis of equality of variances will be carried out, Levene's test. Once the parametricity is confirmed, a comparison of two groups will be made with their respective means or averages using Student's T test for paired or correlated data as well as for independent data, this will be applied for each result variable. It will be determined if there is a difference between the groups with the P value and hypothesis contrast.

Regarding group correlation, the Pearson Correlation statistical test can be used for parametric data.

If the data are non-parametric, the statistical tests to be used as bivariate analysis would be with Medians, Wilcoxon for related or paired samples and Mann Whitney U for independent samples.

**Phase VII: Discussion of results.**

A bibliographic characterization will be determined for each variable compared to similar studies, determining the relationship between the different populations and the effects of the program.

**Phase VIII: Conclusions and/or recommendations**

**Conclusions**

At this point, the results of the research on the population under study will be presented according to the variables evaluated during the project, which must respond to the objectives set at the beginning of the research.

**recommendations**

At the end, with the identification of the admission conditions and the characterization of the population, strategies for a comprehensive intervention are proposed as part of a rehabilitative process. Design a rehabilitation program that allows better benefit for the patient.

**Phase IX: Preparation of the final report**

At the end of the phases, a final report will be prepared, which includes step by step, the points developed throughout the research process, leaving in a summarized and clear way the results obtained in the study and evidence of the product, in this case 1 article submitted.

**ETHICAL CONSIDERATIONS**

The research will take RESOLUTION No. 8430 of October 4, 1993 of the Ministry of Health, which establishes the scientific and administrative standards for health research and the Declaration of Helsinki.

RESOLUTION Nª 8430 in its ARTICLE 9. In which the risk of the investigation is defined as:

risk greater than the minimum since the probabilities of affecting the subject are significant, among which are considered: radiological and microwave studies, studies with the medications and modalities defined in titles III and IV of this resolution, trials with new devices, studies that include surgical procedures, blood collection greater than 2% of the circulating volume in neonates, amniocentesis and other invasive techniques or major procedures, those that use random methods of assignment to therapeutic schemes and those that have control with placebos, among others .

ARTICLE 16: The Informed Consent must present the following information, which will be explained completely and clearly to the research subject or, failing that, to their legal representative, in such a way that they can understand it. This research adopts the requirements established in this article in informed consent. (APPENDIX 1)

Regarding the Declaration of Helsinki, the sections considered relevant for the research will be complied with: general principles, especially paragraphs 6, 7,9. In the section on aspects of research ethics committees, paragraph 23, privacy and confidentiality, paragraph 24, finally emphasizing the section on informed consent in paragraphs 25, 26, 27. The approval of the committee was obtained from the Ethics Committee of the National Sports School University Institution according to Act 17,115 (ANNEX 4), in turn, the endorsement of the participating institution was obtained to carry out the project (ANNEX 5).

It is important to keep in mind that the participant may withdraw from the study at any time without this affecting their provision of cardiac rehabilitation care. For this reason, their responsibility as a participant will end. However, the program will give them the alternative of being able to complete their cardiac rehabilitation program either in outpatient consultation or virtually. (The way that is most convenient for the patient).

The management given to the information obtained and that guarantees confidentiality is: During participation in this research project, absolute confidentiality is guaranteed with the information collected during the process, since this information will be used solely for academic purposes by the main researcher. If you wish, at the end of the study you will be informed of the general results of the research.

| **Fase** | **Activity** | **Year** |
| --- | --- | --- |
| 1 | Research variable determinations  Determination of population under study  Selection of instruments and measurements to be carried out  Intervention protocol design  Obtaining approval from the institutional ethics committee | June 2022 |
| 2 | Linking the population to be studied  Protocol intervention  Information collection | July - December 2022 |
| 3 | Information analysis  Writing final report of the research project  Presentation of products agreed for the call  Socialization of research results | January-November 2023 |

**Bibliographic references**

1. Enfermedades Cardiovasculares. Prevención y control de las enfermedades no transmisibles [Internet]. OMS. 2016 [citado 19 octubre 2021]. Disponible en: https://www.paho.org/es/temas/enfermedades-cardiovasculares. Organización Mundial de la Salud. Estrategia mundial para la prevención y el control de las enfermedades no transmisibles. 2000.
2. Tersalvi G, Winterton D, Cioffi GM, et al. Telemedicine in Heart Failure During COVID-19: A Step Into the Future. Front Cardiovasc Med. 2020;7:612818. doi: 10.3389/fcvm.2020.612818.
3. Piotrowicz E, Pencina MJ, Opolski G, Zareba W, et al. Effects of a 9-Week Hybrid Comprehensive Telerehabilitation Program on Long-term Outcomes in Patients With Heart Failure: The Telerehabilitation in Heart Failure Patients (TELEREH-HF) Randomized Clinical Trial. JAMA Cardiol. 2020;5(3):300-308. doi: 10.1001/jamacardio.2019.5006.
4. Piotrowicz E, Stepnowska M, Leszczyńska-Iwanicka K, et al. Quality of life in heart failure patients undergoing home-based telerehabilitation versus outpatient rehabilitation a randomized controlled study. Eur J Cardiovasc Nurs. 2015;14(3): 256–263. [doi:10.1177/1474515114537023](https://doi.org/10.1177/1474515114537023).
5. Brouwers RWM, van Exel HJ, van Hal JMC, et al. Cardiac telerehabilitation as an alternative to centre-based cardiac rehabilitation. Neth Heart J. 2020;28(9):443-451. doi: 10.1007/s12471-020-01432-y.
6. Ribeiro F, Santos M. Exercise-based cardiac rehabilitation in COVID-19 times: one small step for health care systems, one giant leap for patients. Rev Esp Cardiol (Engl Ed). 2020;73(11):969-970. doi: 10.1016/j.rec.2020.07.002.
7. Maroto-Montero, J., Coello-Cremades, M., Martín-Argomanid, L., Maroto-de-Pablo, M., Gómez-Martínez, M., Paredes-Camargo, C., & de-Pablo-Zarzosa, C. Cardiac rehabilitation in the COVID-19 time. Programs. Results. Arch Cardiol Mex. 2021;91:25-33.
8. Vonk T, Nuijten MAH, Maessen MFH, et al. Identifying Reasons for Nonattendance and Noncompletion of Cardiac Rehabilitation: insights from germany and the netherlands. J Cardiopulm Rehabil Prev. 2021;41(3):153-158. doi: 10.1097/HCR.0000000000000580.
9. Frederix I, Hansen D, Coninx K, et al. Telerehab III: a multi-center randomized, controlled trial investigating the long-term effectiveness of a comprehensive cardiac telerehabilitation program--rationale and study design. BMC Cardiovasc Disord. 2015;15:29. doi: 10.1186/s12872-015-0021-5.
10. McMurray JJ, Adamopoulos S, Anker SD, et al. ESC guidelines for the diagnosis and treatment of acute and chronic heart failure 2012: The Task Force for the Diagnosis and Treatment of Acute and Chronic Heart Failure 2012 of the European Society of Cardiology. Developed in collaboration with the Heart Failure Association (HFA) of the ESC. Eur J Heart Fail 2012; 14: 803–869.
11. Taylor RS, Dalal HM, McDonagh STJ. The role of cardiac rehabilitation in improving cardiovascular outcomes. Nat Rev Cardiol. 2022;19(3):180-194. doi: 10.1038/s41569-021-00611-7.
12. Ades P, Keteyian S, Balady G, et al. Cardiac Rehabilitation Exercise and Self-Care for Chronic Heart Failure. J Am Coll Cardiol HF. 2013 Dec, 1 (6) 540–547. doi:10.1016/j.jchf.2013.09.002
13. Hwang R, Bruning J, Morris NR, Mandrusiak A, Russell T. Home-based telerehabilitation is not inferior to a centre-based program in patients with chronic heart failure: a randomised trial. J Physiother. 2017;63(2):101-107. doi: 10.1016/j.jphys.2017.02.017.
14. Batalik L, Filakova K, Batalikova K, Dosbaba F. Remotely monitored telerehabilitation for cardiac patients: A review of the current situation. World J Clin Cases. 2020;8(10):1818-1831. doi: 10.12998/wjcc.v8.i10.1818.
15. Oscalices MIL, Okuno MFP, Lopes MCBT, Campanharo CRV, Batista REA. Discharge guidance and telephone follow-up in the therapeutic adherence of heart failure: randomized clinical trial. Rev. Latino-Am. Enfermagem. 2019;27:e3159. doi: 10.1590/1518-8345.2484-3159.
16. Bernocchi P, Vitacca M, La Rovere MT, et al. Home-based telerehabilitation in older patients with chronic obstructive pulmonary disease and heart failure: a randomised controlled trial. Age Ageing. 2018;47(1):82-88. doi: 10.1093/ageing/afx146.
17. Bocalini DS, dos Santos L, Serra AJ. Physical exercise improves the functional capacity and quality of life in patients with heart failure. Clinics (Sao Paulo). 2008;63(4):437-42. doi: 10.1590/s1807-59322008000400005.
18. Peng X, Su Y, Hu Z, Sun X, Li X, Dolansky MA, Qu M, Hu X. Home-based telehealth exercise training program in Chinese patients with heart failure: A randomized controlled trial. Medicine (Baltimore). 2018;97(35):e12069. doi: 10.1097/MD.0000000000012069.
19. Pimenta T, Tavares H, Ramos J, Oliveira M, Reis D, Amorim H, Rocha A. Facemasks during aerobic exercise: Implications for cardiac rehabilitation programs during the Covid-19 pandemic. Rev Port Cardiol. 2021 Dec;40(12):957-964. doi: 10.1016/j.repc.2021.01.017.
20. Tam CF, Cheung KS, Lam S, Wong A, Yung A, Sze M, et al. Impact of Coronavirus Disease 2019 (COVID-19) Outbreak on ST-Segment-Elevation Myocardial Infarction Care in Hong Kong, China. Circ Cardiovasc Qual Outcomes. 2020;13(4): 1-3.
21. Tersalvi G, Winterton D, Cioffi GM, et al. Telemedicine in Heart Failure During COVID-19: A Step Into the Future. Front Cardiovasc Med. 2020;7:612818.
22. Kikuchi A, Taniguchi T, Nakamoto K, Sera F, Ohtani T, Yamada T, Sakata Y. Feasibility of home-based cardiac rehabilitation using an integrated telerehabilitation platform in elderly patients with heart failure: A pilot study. J Cardiol. 2021 Jul;78(1):66-71. doi: 10.1016/j.jjcc.2021.01.010.
23. Brown RA. Rehabilitation of patients with cardiovascular diseases. Report of a who expert committee. World Health Organ Tech Rep Ser 1964;270:3-46.
24. López-Jiménez F, Pérez-Terzic C, Zeballos PC, et al . Consenso de Rehabilitación Cardiovascular y Prevención Secundaria de las Sociedades Interamericana y Sudamericana de Cardiología. Rev.Urug.Cardiol. 2013;28(2): 189-224.
25. American Association of Cardiovascular Pulmonary Rehabilitation. Guidelines for Cardiac Rehabilitation and Secondary Prevention Programs. 4th ed. Champaign: Human Kinetics; 2004.
26. Hosseiniravandi M, Kahlaee AH, Karim H, Ghamkhar L, Safdari R. Home-based telerehabilitation software systems for remote supervising: A systematic review. Int J Technol Assess Health Care. 2020;10:1–13.
27. Carrió FB. El modelo biopsicosocial en evolución. Medicina clínica. 2002;119(5):175-9.
28. Osorio MAM. Del Modelo Biomédico al Modelo Biopsicosocial: El desafío pendiente para la fisioterapia en el dolor músculoesquelético crónico. Revista Facultad de Ciencias de la Salud UDES. 2016;3(2):97-101.

**ANNEXES**

**ANNEX 1. INFORMED CONSENT**

**1. INFORMED CONSENT**

| Informed consent is understood as the written agreement by which the research subject, or, where applicable, his or her legal representative, authorizes his or her participation in the research, with full knowledge of the nature of the procedures, benefits, and risks to which he or she will be subjected. , with the capacity for free choice and without any coercion; This phase of the research process is essential, since it allows the participant to be involved as an object of study. This Informed Consent is based on Resolution No. 008430 of October 4, 1993 of the Ministry of Health, which establishes the scientific and administrative standards for health research. | | | | |
| --- | --- | --- | --- | --- |
| 1. El proyecto: | Physical exercise and telephone monitoring mediated by cardiac telerehabilitation in patients with heart failure | | | |
| 1. Researchers: | | | | |
|  | | | | |
| Name (s) | | Last name | ID | Cellphone |
|  | | | | |
| JHONATAN | | BETANCOURT PEÑA | 1130626652 | 33177602534 |
|  | | | | |
|  | | | | |

**INFORMATION ABOUT THE PROJECT**

An investigation is carried out on adherence in a cardiac and pulmonary rehabilitation program in a clinic in Cali.

| Cardiovascular diseases are the leading cause of death and disability worldwide, accounting for approximately 31% (17.9 million) of total deaths each year. The COVID-19 pandemic has led to the total suspension of most cardiac rehabilitation programs at the highest peaks of the spread, forcing people not to leave home, enhancing metabolic conditions and generating greater complications due to a sedentary lifestyle. Physical exercise is an essential component in the rehabilitation of patients with heart failure disease, showing improvements in quality of life, functional capacity, in addition to reducing the mortality rate, number of rehospitalizations, and levels of depression. It is necessary to carry out interventions adapted to the needs of patients who have difficulties traveling to health centers; however, some authors report that remotely oriented exercise could present results similar to those of traditional rehabilitation in a center or clinic, so promoting a cardiac telerehabilitation program together with telephone educational follow-up could cause greater improvements compared to other cardiac rehabilitation and telerehabilitation programs. |
| --- |
| GENERAL OBJECTIVE.  To describe the effects of a physical exercise program in patients with heart failure with different degrees of ventricular dysfunction mediated by telerehabilitation on functional capacity, health-related quality of life and depression.  SPECIFIC OBJECTIVES.   - Sociodemographically and clinically characterize the study population - Determine the effects of a physical exercise program and conventional educational component in patients with heart failure on functional capacity, depression, and health-related quality of life. - Determine the effects of a physical exercise program and conventional educational component mediated by cardiac telerehabilitation in patients with heart failure on functional capacity, depression and health-related quality of life - Determine the effects of a physical exercise program and educational component with telephone follow-up mediated by cardiac telerehabilitation in patients with heart failure on functional capacity, depression and health-related quality of life - Estimate differences in functional capacity, depression, and health-related quality of life between the intervention group and comparison groups. |
| The Procedures that will be used and their purpose are: The research will be carried out with greater than minimal risk, requiring a directed survey, taking paraclinical variables and performing exercise according to the group to which it is linked. If an accident were to occur, the procedure to follow would initially be to provide first aid followed by calling the emergency service of the health service provider entity to which you as a user are affiliated, generating a detailed report to you and, if applicable, your family members about what happened. |
| The present investigation is called greater than minimum risk because an intervention is carried out on physiological variables and includes procedures such as physical exercise, application of questionnaires related to functionality and Health-Related Quality of Life (HRQoL). |
| The Benefits that you will obtain as a participant in this research are: By participating in the research you will obtain direct benefits for yourself, because although you will not have financial remuneration, with your participation we will achieve the objectives of our research, which will possibly be of great benefit. help for your quality of life.. |
| The duration of the participant in the study will be in this research it is estimated to be approximately 12 weeks. |
| Each participant has a medical order to perform cardiac rehabilitation. Upon entering the program, a physiotherapist specializing in cardiopulmonary rehabilitation will recruit potential participants and indicate each procedure. Furthermore, participation in the study is voluntary. Remember that you may withdraw from the study at any time without this affecting your provision of cardiac rehabilitation care. For this reason, your responsibility as a participant will end. However, the program will give you the alternative of being able to complete your cardiac rehabilitation program either in external consultation or virtually. (The way that is most convenient for you) |
| The number of participants included in the study is 156 participants. |
| The management given to the information obtained and that guarantees confidentiality is: During participation in this research project, absolute confidentiality is guaranteed with the information collected during the process, since this information will be used solely for academic purposes by the main researcher. If you wish, at the end of the study you will be informed of the general results of the research. |

1. DECLARACIÓN DEL PARTICIPANTE

Este documento certifica que el participante(s) confirma por escrito su decisión VOLUNTARIA de participar en el presente estudio, después de escuchar todo lo relativo a los objetivos y la metodología que se va a utilizar durante su desarrollo. Se le ha solicitado el consentimiento al tiempo que se le ha explicado que al otorgarlo, puede revocarlo en cualquier momento cuando a juicio del participante o sus familiares lo consideren oportuno sin perjuicio de la atención a su salud y que se garantiza la confidencialidad de la información que sobre su salud se obtenga.

Yo, ______________________________________________________.Conociendo todo lo anterior declaro que:

1. Que toda la información brindada por mí es real y no estoy omitiendo dato alguno que perjudique el desarrollo de la investigación.
2. Tengo derecho a negarme a dar el consentimiento de participar en esta investigación, sin perjuicio de la atención que recibo en esta institución.
3. Se me ha explicado y he comprendido satisfactoriamente la naturaleza y propósito de este estudio.
4. Autorizo a realización de examen clínico o responder las preguntas o el tratamiento que sean necesarias en los cuales el manejo de la confidencialidad, privacidad e identidad serán acordes y permitidas por Ley y no estarán a disposición pública.
5. Tengo derecho a recibir respuesta oportuna y veraz a cualquier pregunta y a que se me aclare cualquier duda acerca de los procedimientos, riesgos, beneficios, y otros asuntos relacionados con la investigación y el tratamiento.
6. Además, se estableció el compromiso por parte de los investigadores de proporcionar información actualizada obtenida durante el estudio, aunque esta pudiera afectar la voluntad del sujeto para continuar participando. Y
7. Se dispondrá de tratamiento médico e indemnización a que legalmente tendría derecho por parte de la institución responsable de la investigación, en el caso de daños que le afecten directamente y que sean causados en el desarrollo de la investigación.
8. Me comprometo a entregar fotocopia del carnet de la EPS y cualquier otro servicio de salud que tenga.
9. **AUTORIZACIÓN Y FIRMAS**

He comprendido todo lo anterior perfectamente y por ello Yo: __________________________________ con documento de identidad: _______________________ expedido en: ______________ doy mi consentimiento para que los estudiantes de ___ semestre de ______________ de la IU Escuela Nacional del Deporte realicen los procedimientos descritos arriba, necesarios durante el desarrollo de este estudio, a juicio de las personas que lo llevan a cabo.

| **Persona mayor de edad participante.** | | | | | | | | | | | | | | | | | | |
| --- | --- | --- | --- | --- | --- | --- | --- | --- | --- | --- | --- | --- | --- | --- | --- | --- | --- | --- |
| Nombre del Participante: | | | | |  | | | | | | | Fecha: | | |  | |  |  |
|  | | | | | | | | | | | | | | | | | | |
| N° Documento de Identidad: | | | | | |  | | | De | |  | | | | | | | |
|  | | | | | | | | | | | | | | | | | | |
| Dirección: | |  | | | | | Firma: | | |  | | | | | | | | |
| **Información del asesor** | | | | | | | | | | | | | | | | | | |
| Nombre del Asesor: | | | |  | | | | | | | | | | | |  | | |
|  | | | | | | | | | | | | | | | | | | |
| Registro: |  | | | | N° Documento de Identidad: | | |  | | | | | De |  | | | | |
|  | | | | | | | | | | | | | | | | | | |
| Firma del Asesor: | | |  | | | |  | | | | | Fecha: | | |  | |  |  |

| **Testigos N° 1** | | | | | | | | | | |
| --- | --- | --- | --- | --- | --- | --- | --- | --- | --- | --- |
| Nombre completo: | |  | | | | | |  | | |
|  | | | | | | | | | | |
| N° Documento de Identidad: | | |  | De |  | | | | | |
|  | | | | | | | | | | |
| Firma |  | | |  | | Fecha: |  | |  |  |
|  | | | | | | | | | | |
| **Testigos N° 2** | | | | | | | | | | |
| Nombre completo: | |  | | | | | |  | | |
|  | | | | | | | | | | |
| N° Documento de Identidad: | | |  | De |  | | | | | |
|  | | | | | | | | | | |
| Firma |  | | |  | | Fecha: |  | |  |  |

**ANNEX 2. MACROPROJECT INFORMATION COLLECTION FORMAT**

| HISTORIA CLÍNICA REHABILITACIÓN CARDIACA | | | | | | | |
| --- | --- | --- | --- | --- | --- | --- | --- |
| NOMBRE | | | | EDAD | | CC | |
| OCUPACIÓN: | TRABAJA: INCAPACIDAD: JUBILADO: NO LABORA: | | | | | | |
| FORMACIÓN: | NIN. PRIMA. SECUN. BACHI TEC. TECNOLO. UNIV. POST. | | | | | | |
| E. CIVIL: | FECHA NACIMIENTO: | | | | | GÉNERO: | |
| NATURAL: | RESIDE: | | | ESTRATO | | TELÉFONO: | |
| FECHA INICIO PROGRAMA: | | | | ENTIDAD: | | CELULAR | |
| EMERGENCIA AVISAR A: | | | | | | TELÉFONO: | |
| MOTIVO DE INGRESO: | | | | | | FECHA | |
| IAM: SI NO | | CARA: ANT INF | | KILLIP: | |  | |
| ANGINA: SI NO ESTABLE: INESTABLE: | | | | | |  | |
| ANGIOPLASTIA: SI NO VASOS: CUALES: | | | | | |  | |
| STENT: SI NO VASOS: CUALES: | | | | | |  | |
| RVM: SI NO VASOS: CUALES: | | | | | |  | |
| ARRITMIA: SI NO CUAL: | | | | | |  | |
| MUERTE SÚBITA: SI NO FECHA: | | | | | |  | |
| ENFERMEDAD CORONARIA: SI NO VASOS CUALES | | | | | |  | |
| EAP: SI NO VASOS: PUENTES: | | | | | |  | |
| CAMBIO VALVULAR: | | | | | |  | |
| CIA: CIV: FONTAN: | | | | | |  | |
| REEMPLAZO AORTA: ASCENDENTE TORÁCICA ABDOMINAL | | | | | |  | |
| ICC: SI NO ESTADO CLASIFICACIÓN FUNCIONAL | | | | | |  | |
| MIOCARDIOPATÍA: SI NO | | | | | |  | |
| TRASPLANTE CARDIACO: SI NO | | | | | |  | |
| SÍNCOPE: SI NO | | | | | |  | |
| MARCAPASO: CARDIODESFIBRILADOR: | | | | | |  | |
| OBSERVACIONES: | | | | | |  | |
| SÍNTOMAS ACTUALES: | | | SI | | NO |  | |
| ANGINA | | |  | |  |  | |
| SÍNCOPE | | |  | |  |  | |
| DISNEA | | |  | |  |  | |
| FATIGA | | |  | |  |  | |
| PALPITACIONES | | |  | |  |  | |
| EDEMA MMII | | |  | |  |  | |
| CLAUDICACIÓN | | |  | |  |  | |
| OTRO | | |  | |  |  | |
| ANTECEDENTES CARDIOVASCULARES | | | SI | | NO | FECHA | OBSERVACIÓN |
| IAM | | |  | |  |  |  |
| ANGINA | | |  | |  |  |  |
| ICC | | |  | |  |  |  |
| STENT | | |  | |  |  |  |
| RVM | | |  | |  |  |  |
| EAP | | |  | |  |  |  |
| RH CARDIACA | | |  | |  |  |  |
| OTRO: | | |  | |  |  |  |

| FACTORES DE RIESGO | | | | | | | | | | | | | | | | | |
| --- | --- | --- | --- | --- | --- | --- | --- | --- | --- | --- | --- | --- | --- | --- | --- | --- | --- |
|  | | | | | | SI | | | | | NO | | | FECHA | | | |
| DISLIPIDEMIA | | | | | |  | | | | |  | | |  | | | |
| HTA | | | | | |  | | | | |  | | |  | | | |
| DM TIPO1 TIPO2 | | | | | |  | | | | |  | | |  | | | |
| OBESIDAD-SOBREPESO | | | | | |  | | | | |  | | |  | | | |
| HIPOTIROIDISMO | | | | | |  | | | | |  | | |  | | | |
| POST MENOPAUSIA | | | | | |  | | | | |  | | |  | | | |
| PESO: TALLA: IMC: PERÍMETRO ABDOMINAL: ÍNDICE CADERA/CINTURA: | | | | | | | | | | | | | | | | | |
| FUMA: SI NO CIGARRILLOS/DÍA: AÑOS: | | | | | | | | | | | | | | | | | |
| HACE CUANTO DEJÓ DE FUMAR: | | | | | | | | | | | | | | | | | |
| ALCOHOL: SI: NO: FRECUENCIA: TIPO: | | | | | | | | | | | | | | | | | |
| ANTECEDENTES FAMILIARES: | | | | | | | | | | | | | | | | | |
| ACTIVIDAD FÍSICA | | | | | | | | | | | | | | | | | |
| TIPO | | | | | | INTENSIDAD | | | | | DURACIÓN MIN | | | FRECUENCIA SEMANA | | | |
| CAMINATA | | | | | | LEVE | | | | | MENOR 15 | | | 1 | | 6 | |
| TROTE | | | | | | MODERADA | | | | | 15-30 | | | 2 | | 7 | |
| BICICLETA | | | | | | VIGOROSA | | | | | 30-45 | | | 3 | |  | |
| NATACIÓN | | | | | |  |  |  |  |  | 45-60 | | | 4 | |  | |
| OTRO | | | | | |  | | | | | MAYOR 60 | | | 5 | |  | |
| SEDENTARIO | | | | | | POCO ACTIVO | | | | | ACTIVO | | | MUY ACTIVO | | ENTRENADO | |
| LABORATORIOS | | | | | | | | | | | | | | | | | |
| FECHA | | | |  | |  | | | | |  | | |  | |  | |
| COLESTEROL | | | |  | |  | | | | | HB | | |  | |  | |
| HDL | | | |  | |  | | | | | HCTO | | |  | |  | |
| LDL | | | |  | |  | | | | | K | | |  | |  | |
| TRIGLICÉRIDOS | | | |  | |  | | | | | NA | | |  | |  | |
| GLICEMIA | | | |  | |  | | | | | CREATININA | | |  | |  | |
| BUN | | | |  | |  | | | | | TSH | | |  | |  | |
| INR | | | |  | |  | | | | | AC ÚRICO | | |  | |  | |
| EXÁMENES PARACLÍNICOS | | | | | | | | | | | | | | | | FECHA | |
| ECOCARDIOGRAMA | | | | | FE: | | | | | | | VÁLVULAS: | | | |  | |
| CONTRACTILIDAD | | | | | | | | | | | | OBSERVACIONES | | | | | |
| PRUEBA DE ESFUERZO | | | | | TIPO | | | | FC MÁX | | | TA MÁX | | | CF | METS | |
| ISONITRILOS | | SI | | | NO | | | | POSITIVA | | | NEGATIVA | | | FE: | CARA: | |
| OTRO: | | | | | | | | | | | | | | | | | |
| CATETERISMO: | | FE: | | | OBSERVACIONES | | | | | | | | | | | | |
| AMA – AS CSM | | | | | | | | | | | | | | | | | |
| A1 | A2 | | | | | | A3 | | | B | | | C | | | | D |
| RIESGO DE EVENTOS | | | | | | | | | | | | | | | | | |
| BAJO | | | | | | | MODERADO | | | | | | ALTO | | | | |
| RIESGO PROGRESIÓN ENFERMEDAD AACVPR | | | | | | | | | | | | | | | | | |
| BAJO | | | | | | | MODERADO | | | | | | ALTO | | | | |
| RIESGO DE EVENTOS CORONARIOS A 10 AÑOS FRAMINGHAM: % | | | | | | | | | | | | | | | | | |
| BAJO | | | LEVE | | | | | MODERADO | | | | ALTO | | | | MUY ALTO | |

| EVALUACIÓN: PRUEBA DE EJERCICIO SUBMÁXIMA | | |
| --- | --- | --- |
| NOMBRE: | | DOCUMENTO: |
| TIPO DE PRUEBA | TC6M | |
| FECHA |  |  |
| FR INICIO |  |  |
| SPO2 INICIO |  |  |
| FC INICIO |  |  |
| TA INICIO |  |  |
| BORG INICIO |  |  |
| FATIGA MMII INICIO |  |  |
| DISTANCIA |  |  |
| FR FINAL |  |  |
| SPO2 FINAL |  |  |
| FC FINAL |  |  |
| TA FINAL |  |  |
| BORG FINAL |  |  |
| FATIGA MMII FINAL |  |  |
| HADS ANSIEDAD |  |  |
| HADS DEPRESIÓN |  |  |
| % GRASA |  |  |
| %AGUA |  |  |
| %MASA |  |  |
| TALLA |  |  |
| PESO |  |  |
| IMC |  |  |
| PERÍMETRO ABDOMINAL |  |  |
| DASI |  |  |
| PHQ9 |  |  |
| OBSERVACIONES: | | |

**ANNEX 3. INTERVENTION PROTOCOLS**

**Intervention Group: Physical exercise program and conventional educational component with telephone follow-up**

The program will be assisted by virtual technology and consists of performing physical exercise at home supervised by a physiotherapist specializing in cardiac and pulmonary rehabilitation through the “Google Meet” platform, for 60 minutes, 3 times a week for a period of 12 weeks. . Each participant will be given a polar FT4 frequency monitor to record their HR, an OMRON digital blood pressure monitor to monitor blood pressure, and the conventional Borg scale will be used to perceive effort during exercise.

The sessions will be held on the virtual platform Google Meet, for which groups of 6 people or less will be formed, at different times in the morning and in which each of the people will be instructed to perform exercise led by a physiotherapist based in the initial assessment previously carried out.

Before each session, people must rest for 15 minutes in a seated position and subsequently take their vital signs (HR and blood pressure) and perception of effort (Borg) for themselves; which will be taken again during (strength and aerobic capacity training) and at the end of the exercise session.

Each session will begin with a 10-minute warm-up performing self-loading activities and position changes. The central phase will be divided into 2 moments, initially, people will perform 20 minutes of muscle strengthening with dumbbells, with intensities of 50% of the RM (11/20 according to the Borg scale), which will increase progressively according to the capabilities of each one at a maximum of 70% of the RM (15/20 according to the Borg scale), distributed between 3 and 4 series for 12 to 15 repetitions, muscular strengthening of the lower limbs with self-loading will also be performed. For its part, the second moment will consist of 25 minutes of aerobic exercise with an intensity of 50% of the HRmax (11/20 according to the Borg scale), progressively increasing every 4 weeks up to a maximum of 70% (15/20 according to the Borg scale). Borg), through walking at home, step or stationary bike, depending on the resources each participant has.

In addition, the cooling phase will be carried out for 5 minutes, which will consist of walking below 50% of HRMax. Likewise, using the conventional Borg effort perception scale, those people who do not reach the maximum intensity of heart rate due to the use of beta blockers will be monitored, maintaining a Borg score of 11 – 15.

The individual and group educational component will be carried out once a week via virtual means with an estimated duration of 30 minutes each. It will be carried out by properly trained health professionals addressing topics related to: Knowledge of the disease, use of medications, warning signs, anxiety management, relaxation techniques, exercises at home, sexual relations and adequate nutrition.

Additionally, telephone calls will be made 3 times a week as a monitoring method to obtain information from each person about their heart rate and perception of effort compared to the activities carried out, where they will be encouraged to perform exercises at home on days when they do not perform cardiac telerehabilitation. (previously trained personnel outside the research)

**Control group 1: Physical exercise program and conventional educational component mediated by cardiac telerehabilitation**

The program will be assisted by virtual technology and consists of performing physical exercise at home supervised by a physiotherapist specializing in cardiac and pulmonary rehabilitation through the “Google Meet” platform, for 60 minutes, 3 times a week for a period of 12 weeks. . Each participant will be given a polar FT4 frequency monitor to record their HR, an OMRON digital blood pressure monitor to monitor blood pressure, and the conventional Borg scale will be used to perceive effort during exercise.

The sessions will be held on the virtual platform Google Meet, for which groups of 6 people or less will be formed, at different times in the morning and in which each of the people will be instructed to perform exercise led by a physiotherapist based in the initial assessment previously carried out.

Before each session, people must rest for 15 minutes in a seated position and subsequently take their vital signs (HR and blood pressure) and perception of effort (Borg) for themselves; which will be taken again during (strength and aerobic capacity training) and at the end of the exercise session.

Each session will begin with a 10-minute warm-up performing self-loading activities and position changes. The central phase will be divided into 2 moments, initially, people will perform 20 minutes of muscle strengthening with dumbbells, with intensities of 50% of the RM (11/20 according to the Borg scale), which will increase progressively according to the capabilities of each one at a maximum of 70% of the RM (15/20 according to the Borg scale), distributed between 3 and 4 series for 12 to 15 repetitions, muscular strengthening of the lower limbs with self-loading will also be performed. For its part, the second moment will consist of 25 minutes of aerobic exercise with an intensity of 50% of the HRmax (11/20 according to the Borg scale), progressively increasing every 4 weeks up to a maximum of 70% (15/20 according to the Borg scale). Borg), through walking at home, step or stationary bike, depending on the resources each participant has.

In addition, the cooling phase will be carried out for 5 minutes, which will consist of walking below 50% of HRMax. Likewise, using the conventional Borg effort perception scale, those people who do not reach the maximum intensity of heart rate due to the use of beta blockers will be monitored, maintaining a Borg score of 11 – 15.

The individual and group educational component will be carried out once a week via virtual means with an estimated duration of 30 minutes each. It will be carried out by properly trained health professionals addressing topics related to: Knowledge of the disease, use of medications, warning signs, anxiety management, relaxation techniques, exercises at home, sexual relations and adequate nutrition.

**Control group 2: Physical exercise program and conventional educational component**

The program will be attended in person in the clinic's cardiac rehabilitation program and consists of performing physical exercise supervised by a physiotherapist specializing in cardiac and pulmonary rehabilitation, for 60 minutes, 3 times a week for a period of 12 weeks.

Each session will begin with a 10-minute warm-up performing self-loading activities and position changes. The central phase will be divided into 2 moments, initially, participants will perform 20 minutes of muscle strengthening with dumbbells, with intensities of 50% of the RM (11/20 according to the Borg scale), which will increase progressively according to the capabilities of each one at a maximum of 70% of the RM (15/20 according to the Borg scale), distributed between 3 and 4 series for 12 to 15 repetitions, muscular strengthening of the lower limbs with self-loading will also be performed. For its part, the second moment will consist of 25 minutes of aerobic exercise with an intensity of 50% of the HRmax (11/20 according to the Borg scale), progressively increasing every 4 weeks up to a maximum of 70% (15/20 according to the Borg scale). Borg), through walking on a treadmill or stationary bicycle.

In addition, the cooling phase will be carried out for 5 minutes, which will consist of walking below 50% of HRMax. Likewise, using the conventional Borg effort perception scale, those people who do not reach the maximum intensity of heart rate due to the use of beta blockers will be monitored, maintaining a Borg score of 11 – 15.

The individual and group educational component will be carried out once a week with an estimated duration of 30 minutes each in person. It will be carried out by properly trained health professionals addressing topics related to: Knowledge of the disease, use of medications, warning signs, anxiety management, relaxation techniques, exercises at home, sexual relations and adequate nutrition.
